# Supplementary material for: Therapeutic effects of TM4SF5-targeting chimeric and humanized monoclonal antibodies in hepatocellular and colon cancer models
Source: Mol Ther Oncolytics. 2022 Jan 31;24:452–66. doi: 10.1016/j.omto.2022.01.006 (PMC8841960; doi:10.1016/j.omto.2022.01.006)

OMTO, Volume *24*

Supplemental information

Therapeutic effects of TM4SF5-targeting chimeric and humanized monoclonal antibodies in hepatocellular and colon cancer models

Dongjoon Ko, Eunmi Kim, Eun-Ae Shin, Seo Hee Nam, Junghwa Yoon, Jin-Sook Lee, Yunhee Lee, Sora Park, Kyungsoo Ha, So-Young Choi, Jung Weon Lee, and Semi Kim

Supplementary Figure Legends

Supplementary Figure S1. (A) HT-29 cells were transfected with siRNA against TM4SF5 for 48 h and then immunostained with Ab27 (5 μg/ml) (green). Cell nuclei were counterstained with DAPI (blue). Scale bar, 50 μm. (B) Effect of STAT3 suppression on cell proliferation. Cells were transfected with siRNA against STAT3 for 48 or 72 h, lysed for immunoblot analysis to confirm suppression of STAT3 (upper), and subjected to cell proliferation assays (lower). Values represent means ± SD. $^{**}P < 0.01$; $^{***}P < 0.001$. (C) Scatter plots examining TM4SF5 mRNA expression (x-axis) and phosphorylated STAT3 at Tyr705 (y-axis) from liver hepatocellular carcinoma data (TCGA, PanCancer Atlas). Correlation was statistically analyzed using the Pearson test. The equation was automatically generated using the cBioPortal webpage tool.

Supplementary Figure S2. SNU449T_7-luc (stably overexpressing TM4SF5 and luciferase) cells (1×10^6) were injected subcutaneously into the backs of mice. On day 14, Ab27 (100 μg/mouse) was injected intraperitoneally at 2 or 3 day intervals for 2 weeks (total 7 times). (A) At 14 and 31 days after cell injection, bioluminescence images were saved. (B) Total bioluminescence flux over 2 week treatment was acquired using an IVIS Luminar imaging system. (C) Body weights of injected mice. Values represent means ± standard deviation (SD). $^{**}P < 0.01$.

Supplementary Figure S3. Ab27 inhibits colon cancer growth in a xenograft model. (A-C, E) HT-29 cells (2.5×10^6) were subcutaneously injected into the flanks of mice. Ab27 (142 μg/mouse) was i.p. injected into mouse (total of six injections). (A) Upper: Tumor volume

(length × width^2/2). Values of the maximum and minimum in each group were excluded from the mean calculation. Middle: Body weight of injected mice. Lower: Photos of tumor-bearing mice on day 33. (B) Ki67 staining of tumor sections was performed to measure the level of cell proliferation. Representative images are shown. Scale bar, 100 μm. (C) Cell death area was measured from (B) using ImageJ. Values represent means ± SD. *$P < 0.05$; **$P < 0.01$. P-value is shown on the graph (A). (D) HT-29 cells were incubated with Ab27 (25 μg/ml) for 48 h under suspension culture conditions, and then stained with annexin V and PI for flow cytometry. HA6 was used as a negative control antibody. (E) Immunoblot analysis of tumor extracts from (A). Densitometric quantification of bands on the immunoblot was performed using GAPDH as a loading control except that phosphorylated STAT3 and ERK1/2 were normalized against the corresponding total protein. Values of the maximum and minimum per group were excluded for the mean calculation. Values represent means ± SD. *$P < 0.05$; **$P < 0.01$.

Supplementary Figure S4. (A, B) Purified recombinant antigen proteins [human EC2-mouse Fc (hEC2-mFc) and mouse EC2-mouse Fc (mEC2-mFc)] (A) and purified Ab27 scFv-6×His-HA form (B) were analyzed by SDS-PAGE followed by Coomassie brilliant blue staining. R, reducing; NR, non-reducing condition. (C) Antigen-binding ELISA. Ninety-six-well immunoplates were coated with purified hEC2-mFc or mEC2-mFc (100 ng/well), and blocked with 2% BSA. Ab27 (scFv-6×His-HA form; serial diluted) was added, followed by HRP-conjugated anti-HA. Color was developed with TMB substrate solution and absorbance was measured at 450 nm.

Supplementary Figure S5. (A) Schematic representation of chimeric (Ab27) and humanized (Ab27-hz9) monoclonal antibodies of scFv-Fc format. C domain, constant domain; V domain, variable domain; VH, heavy chain variable domain; VL, light chain variable domain. (B) *In silico* immunogenicity analysis. Identification of immunogenic sequences was performed using the IEDB MHC II prediction server (Kim, Y., et al. (2012). Immune epitope database analysis resource. Nucleic Acids Res. 40, W525–W530), which employs a consensus approach combining NN-align, SMM-align, CombLib, and Sturniolo method. The results from IEDB prediction were visualized using the web-based analysis system in KBIOHealth (Osong, Korea). In the visualized results, colors indicate the degree of immunogenicity of an amino acid sequence, determined by the percentile rank. Immunogenic sequences on the right heat map are represented by 25%, 35%, and 50% cut-offs for low (P10, yellow), medium (P5, orange), and high (P1, red), respectively. VH, heavy chain variable domain; VL, light chain variable domain.

Supplementary Figure S6. Purified Ab27-hz9 was analyzed by SDS-PAGE, followed by Coomassie brilliant blue staining. Human IgG was loaded for comparison. R, reducing; NR, non-reducing condition.

Supplementary Figure S7. *In vivo* tumor targeting of Ab27 and Ab27-hz9 in an endogenous TM4SF5-expressing liver cancer xenograft. SNU-398 cells were subcutaneously injected into the flanks of nude mice to generate tumor-bearing mice. DyLight 755-labeled Ab27, Ab27-hz9, or normal human IgG was injected into the tail vein of tumor-bearing mice, and fluorescence was measured after 96 h. (A) Whole bodies of mice. (B)

Dissected mice at 96 h. Arrow indicates tumor mass. (C) Total fluorescence flux of tumor mass. Values represent means ± SD. $^{**}P < 0.01$.

Supplementary Figure S1. Ko et al.

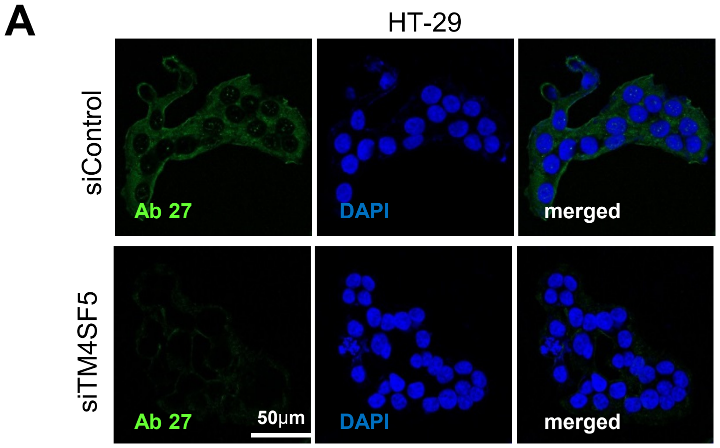

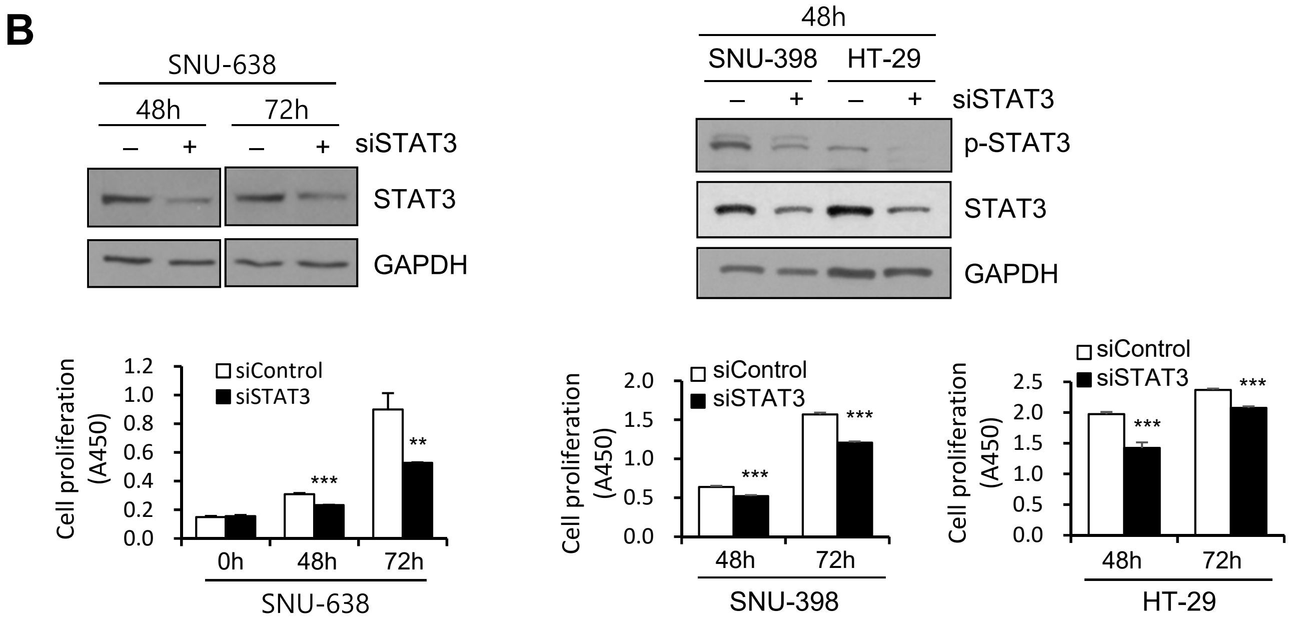

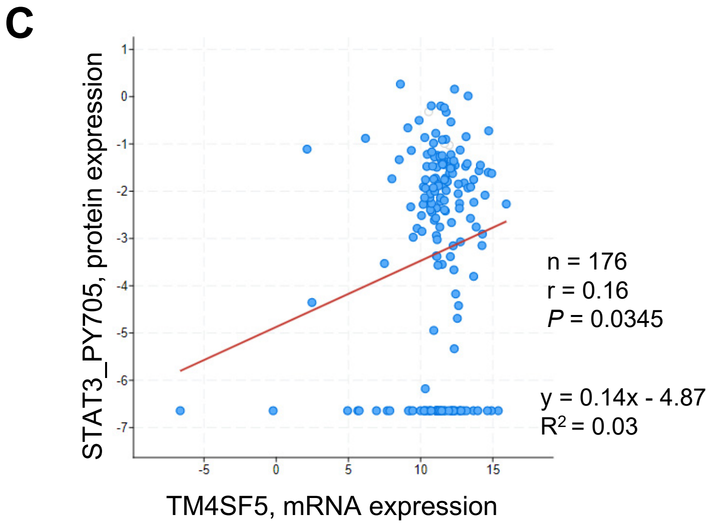

Supplementary Figure S2. Ko et al.

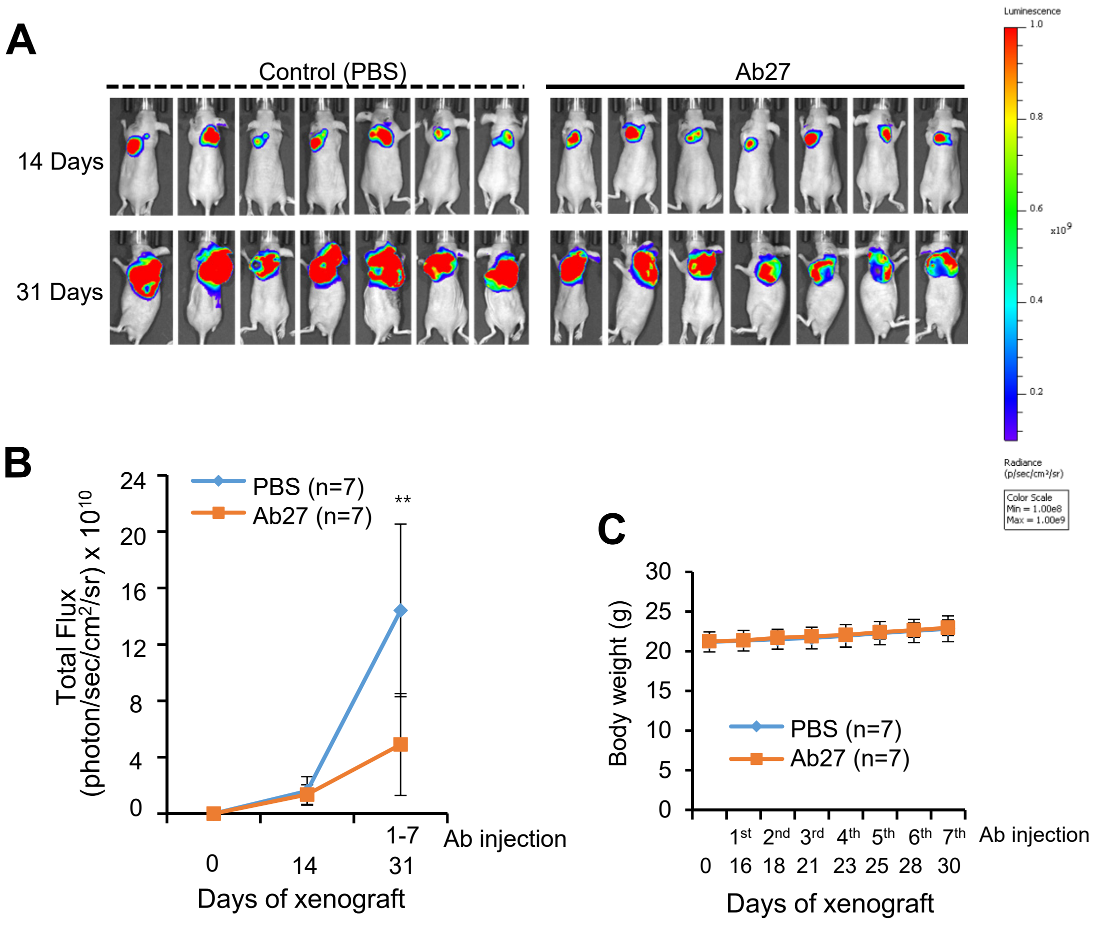

Supplementary Figure S3. Ko et al.

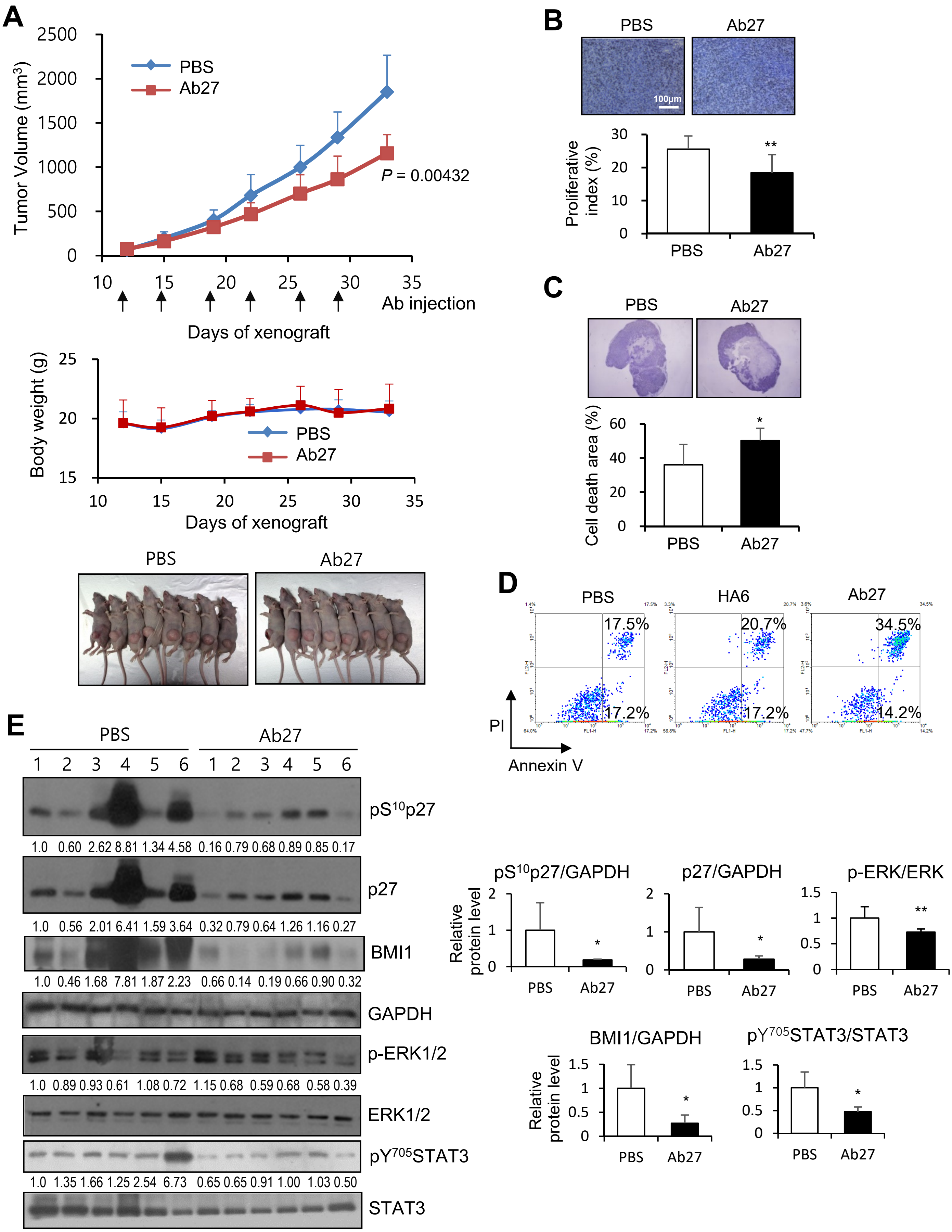

Supplementary Figure S4. Ko et al.

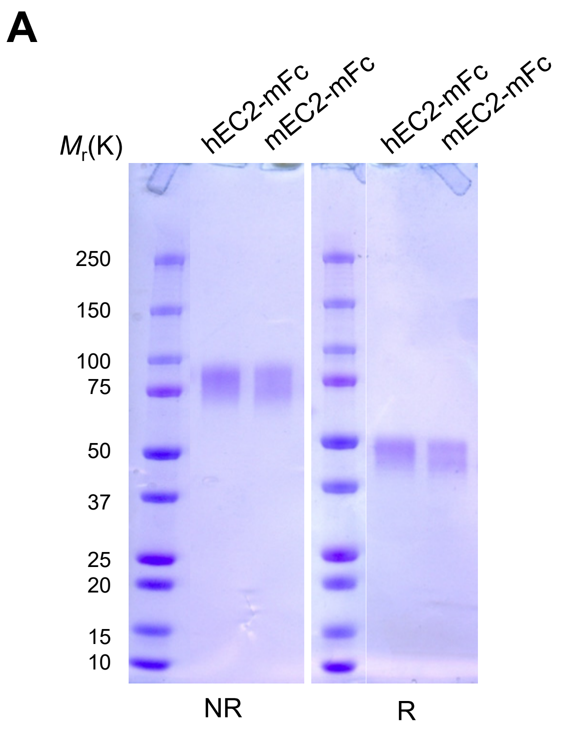

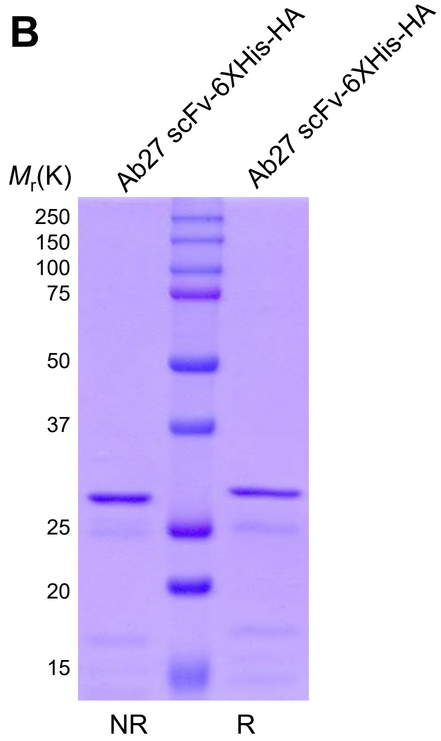

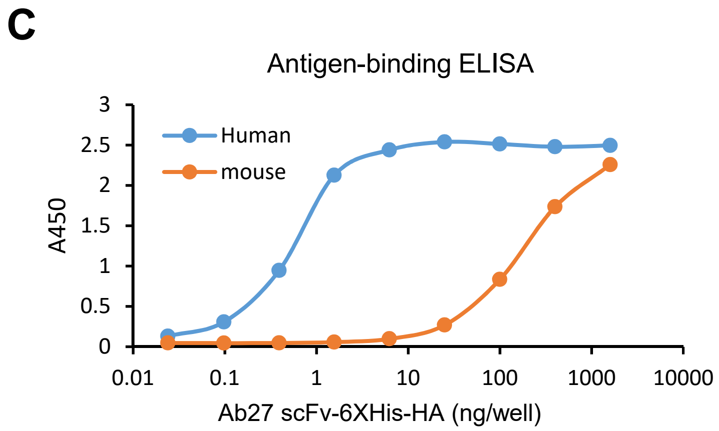

Supplementary Figure S5. Ko et al.

A

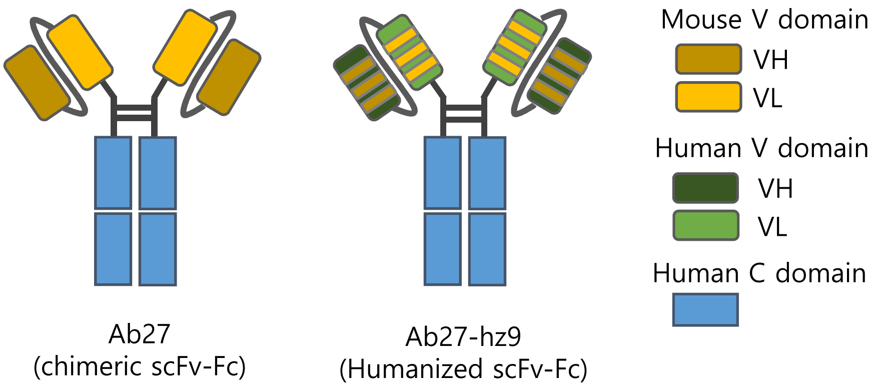

B

In silico immunogenicity analysis

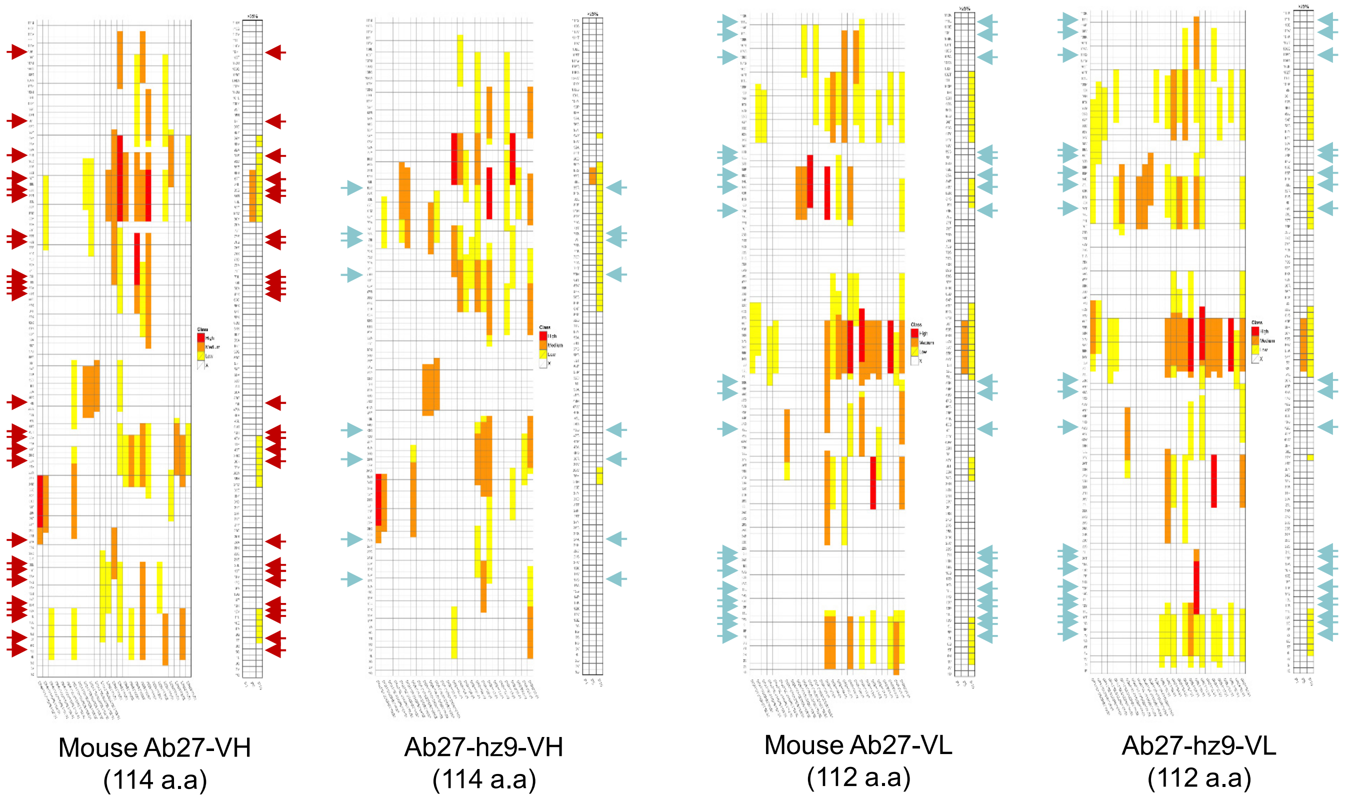

Supplementary Figure S6. Ko et al.

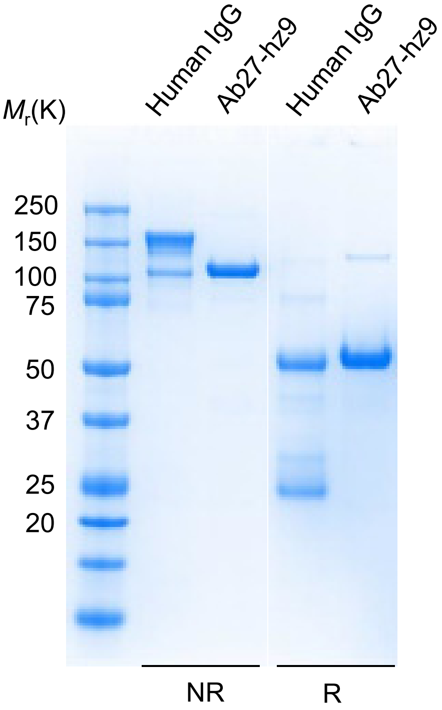

Supplementary Figure S7. Ko et al.

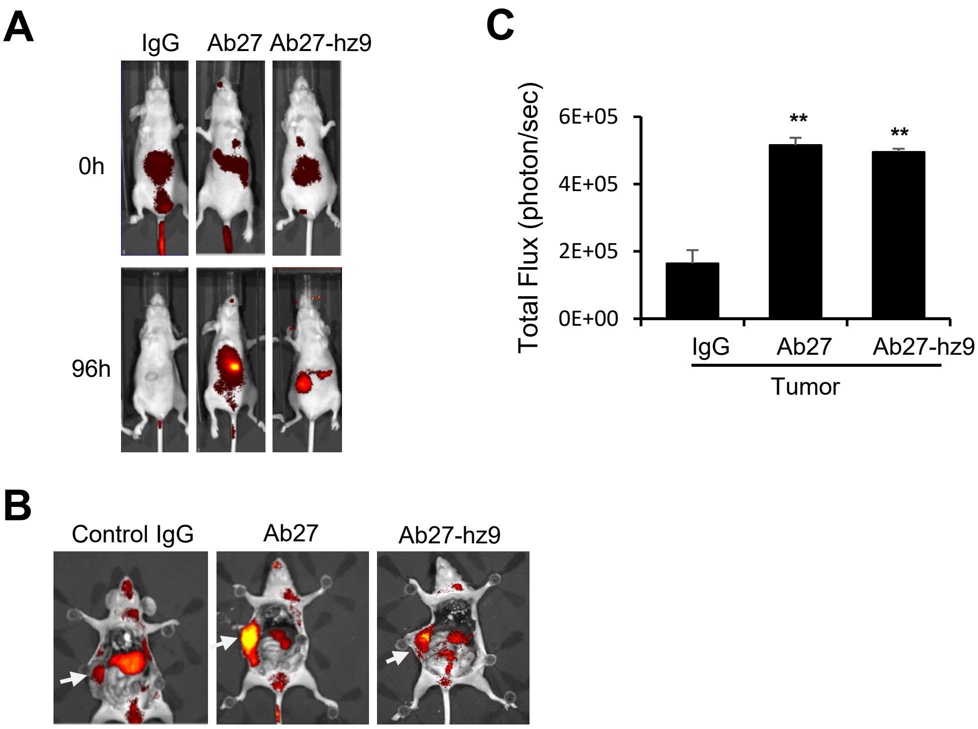

Supplement: Document S1. Figures S1–S7 [file mmc1.pdf]
