# Supplementary material for: Therapeutic effects of TM4SF5-targeting chimeric and humanized monoclonal antibodies in hepatocellular and colon cancer models
Source: Mol Ther Oncolytics. 2022 Jan 31;24:452–66. doi: 10.1016/j.omto.2022.01.006 (PMC8841960; doi:10.1016/j.omto.2022.01.006)

Molecular Therapy
Oncolytics
Original Article

Therapeutic effects of TM4SF5-targeting chimeric and humanized monoclonal antibodies in hepatocellular and colon cancer models

Dongjoon Ko,[1,2] Eunmi Kim,[3] Eun-Ae Shin,[3] Seo Hee Nam,[3] Junghwa Yoon,[1] Jin-Sook Lee,[1] Yunhee Lee,[1] Sora Park,[4] Kyungsoo Ha,[5] So-Young Choi,[6] Jung Weon Lee,[3] and Semi Kim[1,2]

[1]Immunotherapy Research Center, Korea Research Institute of Bioscience and Biotechnology, 125 Gwahak-ro, Yuseong-gu, Daejon 34141, Korea; [2]Department of Functional Genomics, Korea University of Science and Technology, Daejon 34113, Korea; [3]Department of Pharmacy Research Institute of Pharmaceutical Sciences College of Pharmacy, Seoul National University, 1 Gwanak-ro, Gwanak-Gu, Seoul 08826, Korea; [4]Antibody Drug Team at New Drug Development Center, Osong Medical Innovation Foundation, Osong 28160, Korea; [5]Drug Efficacy Evaluation Team at New Drug Development Center, Osong Medical Innovation Foundation, Osong 28160, Korea; [6]Protein Drug Team at New Drug Development Center, Osong Medical Innovation Foundation, Osong 28160, Korea

The transmembrane 4 L six family member 5 (TM4SF5) is aberrantly expressed in hepatocellular and colorectal cancers, and has been implicated in tumor progression, suggesting that it could serve as a novel therapeutic target. Previously, we screened a murine antibody phage-display library to generate a novel monoclonal antibody, Ab27, that is specific to the extracellular loop 2 of TM4SF5. In this study, we evaluated the effects of chimeric Ab27 using cancer cells expressing endogenous TM4SF5 or stably overexpressing TM4SF5 *in vivo* and *in vitro*. Monotherapy with Ab27 significantly decreased tumor growth in liver and colon cancer xenograft models, including a sorafenib-resistant model, and decreased the phosphorylation of focal adhesion kinase (FAK), $p27^{Kip1}$, and signal transducer and activator of transcription 3 (STAT3). No general Ab27 toxicity was observed *in vivo*. Combination treatment with Ab27 and sorafenib or doxorubicin exerted higher antitumor activity than monotherapy. In addition, we humanized the Ab27 sequence by the complementarity-determining region (CDR) grafting method. The humanized antibody Ab27-hz9 had reduced immunogenicity but exhibited target recognition and antitumor activity comparable with those of Ab27. Both Ab27 and Ab27-hz9 efficiently targeted tumor cells expressing TM4SF5 *in vivo*. These observations strongly support the further development of Ab27-hz9 as a novel therapeutic agent against liver and colorectal cancers.

INTRODUCTION

Hepatocellular carcinoma (HCC), the most common primary liver cancer, is the second leading cause of cancer-related death.[1,2] The prognosis of HCC is poor, and systemic treatment options are limited. Sorafenib, a multi-kinase inhibitor, is the main systemic therapeutic option in patients with advanced HCC, but the high resistance rate and significant side effects of the drug have significantly limited the benefit of sorafenib therapy.[3] To overcome sorafenib resistance, it will be necessary to identify new therapeutic targets and develop specific therapeutics for advanced HCC. Colorectal cancer (CRC) is among the most lethal and prevalent malignant tumors around the world.[1,4] As with HCC, the prognosis of CRC is not satisfying, especially for patients with metastasis, although there have been advances in treatment options, including the anti-epidermal growth factor receptor (EGFR) antibody cetuximab and the anti-angiogenesis antibody bevacizumab.[4] Effective therapies for both advanced liver cancer and CRC patients are urgently required.

The transmembrane 4 L six family member 5 (TM4SF5) is a member of the transmembrane 4 L six family, similar to the tetraspanin family.[5] TM4SF5 is a cell surface protein with 4 transmembrane domains and 2 extracellular loops. TM4SF5 is highly expressed in diverse cancers, including liver, colon, pancreatic, and esophageal cancers.[6–8] Previously, we reported that TM4SF5 plays a critical role in HCC development and metastasis; specifically, it mediates the epithelial-mesenchymal transition (EMT) and proliferation by phosphorylating FAK/c-Src and $p27^{Kip1}$ and upregulating the expression of $p27^{Kip1}$, and angiogenesis by upregulating the expression of vascular endothelial growth factor (VEGF).[7,9] It also promotes self-renewal of circulating tumor cells through its association with CD44 and subsequent activation of the c-Src/STAT3/TWIST1/BMI1 pathway.[10] TM4SF5 crosstalks with integrins α2, α5, and β1 and EGFR to mediate cell migration, tumorigenesis, and drug resistance.[9,11] The interactions of TM4SF5 with integrin α2β1,[12] EGFR,[11] and CD44[10] are probably mediated by its extracellular

Received 27 August 2021; accepted 21 January 2022;
https://doi.org/10.1016/j.omto.2022.01.006.

Correspondence: Jung Weon Lee, PhD, Department of Pharmacy, College of Pharmacy, Seoul National University, 1 Gwanak-ro, Gwanak-Gu, Seoul 08826, Korea
E-mail: jwl@snu.ac.kr
Correspondence: Semi Kim, PhD, Immunotherapy Research Center, Korea Research Institute of Bioscience and Biotechnology, 125 Gwahak-ro, Yuseong-gu, Daejon 34141, Korea
E-mail: semikim@kribb.re.kr

 © 2022 The Authors.
This is an open access article under the CC BY license (http://creativecommons.org/licenses/by/4.0/).

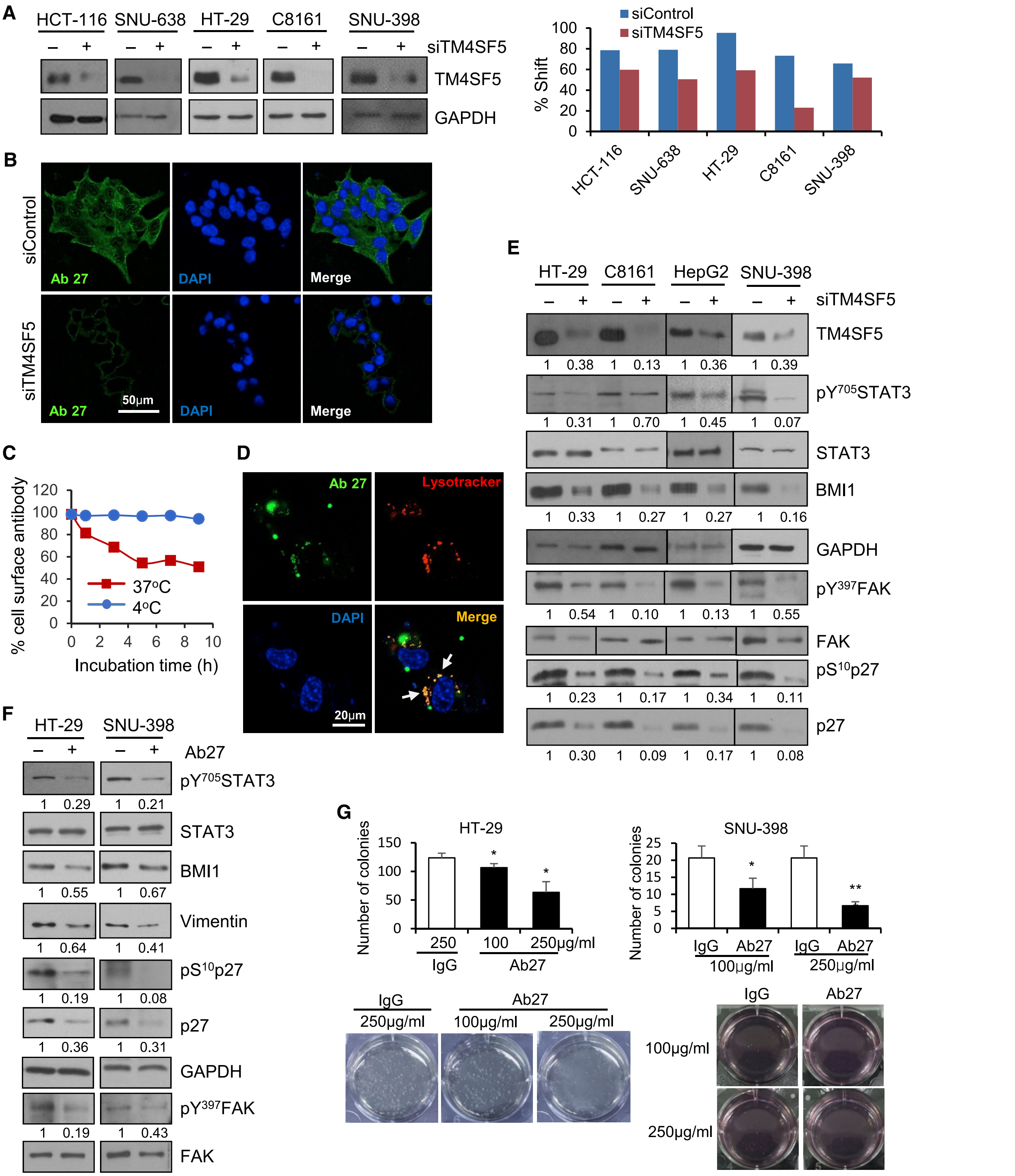

Figure 1. Ab27 inhibits cancer cell growth by suppressing TM4SF5-mediated STAT3 phosphorylation

(A) Cells were transfected with siRNA against *TM4SF5* for 48 h before lysis for immunoblot analysis with rabbit anti-TM4SF5 (in-house) (left) and flow cytometry analysis with Ab27 (right). The extent of a shift in the fluorescence signal compared to control staining, representing binding activity of antibody, is shown as a graph (right). (B) Cells were transfected with siRNA against *TM4SF5* for 48 h and then immunostained with Ab27 (5 μg/mL) (green). Cell nuclei were counterstained with DAPI (blue). Scale bar, 50 μm. (C) Internalization analysis. HCT-116 cells were incubated with Ab27 (0.3 μg/sample) for 45 min at 4°C, washed to remove unbound antibodies, and then either warmed to 37°C to allow internalization or maintained at 4°C for the indicated periods. Cells were stained with FITC-conjugated anti-human IgG and analyzed by flow cytometry.

(legend continued on next page)

loop 2 (EC2), which may play a critical role in the promotion of tumors, as suggested by studies of the effects of point mutations of *N*-glycosylated residues within the EC2 and blocking of the EC2 domain with an anti-TM4SF5 compound, the synthetic chalcone derivative 4′-(*p*-toluenesulfonylamido)-4-hydroxychalcone (TSAHC).[13] These observations suggest that TM4SF5 could be targeted for anticancer therapy using, for example, a TM4SF5-targeting monoclonal antibody.

Previously, we identified novel monoclonal antibodies specific to the EC2 domain of human TM4SF5 by screening a murine antibody (single-chain variable fragment; scFv) phage-display library.[14] One of the clones, Ab27, a chimeric antibody of scFv fused to the Fc domain of human immunoglobulin G1 (IgG1) (the scFv-Fc format[15]), reacted with recombinant TM4SF5 EC2 protein and naive TM4SF5 on the cell surface and was effective in inhibiting cancer cell invasion and proliferation. Ab27 mediated antibody-dependent cell-mediated cytotoxicity. Intratumoral injection of Ab27 efficiently decreased ectopic TM4SF5-expressing tumor growth *in vivo*.[14]

In this study, we evaluated the therapeutic potential of the Ab27 chimeric monoclonal antibody against liver and colon cancer cells expressing endogenous levels of TM4SF5 or stably overexpressing the protein *in vitro* and *in vivo*. Furthermore, we generated a humanized monoclonal antibody, Ab27-hz9, and evaluated its reactivity *in vitro* and *in vivo*. Ab27 suppressed the tumor growth in liver and colon cancer xenograft models, including sorafenib-resistant HCC, and decreased the phosphorylation of FAK, $p27^{Kip1}$, and STAT3. Combination treatment with Ab27 and sorafenib or doxorubicin resulted in higher antitumor activity than monotherapy. Ab27-hz9 exhibited target recognition and antitumor activity comparable with those of Ab27, indicating that Ab27-hz9 could serve as a starting platform for the further development of clinical treatments for liver cancer and CRC.

RESULTS

Ab27 suppresses TM4SF5-mediated STAT3 activation to inhibit cancer cell growth

To determine whether Ab27 specifically recognizes endogenous TM4SF5 on the cancer cell surface, we performed flow cytometry of several types of cancer cells transiently transfected with *TM4SF5*-specific or negative control small interfering RNA (siRNA). As shown in Figure 1A, Ab27 bound to HCT-116, HT-29 (colon), SNU-398 (liver), SNU-638 (stomach), and C8161 (melanoma) cancer cells expressing endogenous TM4SF5, and suppression of TM4SF5 expression by siRNA decreased Ab27 binding to these cells, confirming the specificity of Ab27. Similarly, immunofluorescence staining revealed that Ab27 substantially stained the cell membrane and cytoplasm of HepG2 (Figure 1B) and HT-29 (Figure S1A) cells, but not those of TM4SF5-suppressed cells. In addition, when HCT-116 cells were incubated with Ab27 to allow internalization, the residual level of cell surface-bound Ab27 was substantially reduced (Figure 1C), indicating that Ab27 was internalized after binding to endogenous TM4SF5 on the cell surface, thereby decreasing the cell-surface level of TM4SF5. This is consistent with our previous observations of ectopically TM4SF5-overexpressing SNU-449Tp cells.[14] In addition, treatment of SNU-449Tp cells with DyLight 488-conjugated Ab27 for 3 h followed by staining with LysoTracker to label lysosomes revealed that Ab27 was localized to lysosomes (Figure 1D), indicating rapid internalization and lysosomal targeting of Ab27.

Previously, we observed that TM4SF5 activates STAT3 in HCC cells.[10] STAT3 facilitates cancer cell proliferation and survival, tumor spheroid formation, and metastasis.[16] Knockdown of STAT3 in SNU-638, SNU-398, and HT-29 cells decreased cell proliferation (Figure S1B), confirming the role of STAT3 in cell proliferation. In SNU-398, HT-29, HepG2, and C8161 cells, the phosphorylation of STAT3 and subsequent expression of BMI1 were reduced following the suppression of TM4SF5 expression (Figure 1E), consistent with the results of our previous studies.[10] In addition, analysis of The Cancer Genome Atlas (TCGA)-generated liver hepatocellular carcinoma data (TCGA, PanCancer Atlas) revealed that *TM4SF5* mRNA expression was positively correlated with the phosphorylation of STAT3 at Tyr705 (Figure S1C). Ab27 decreased the phosphorylation of STAT3 and expression of BMI1 in SNU-398 and HT-29 cells (Figure 1F), and it significantly decreased the anchorage-independent growth of SNU-398 and HT-29 cells in a dose-dependent manner (Figure 1G). These results indicate that Ab27 suppresses TM4SF5-mediated STAT3 activation, contributing to the reduction of cancer cell growth. Consistent with our previous results,[7,14] the phosphorylation of FAK and $p27^{Kip1}$ and the expression of $p27^{Kip1}$ were also reduced by TM4SF5 knockdown (Figure 1E) and Ab27 (Figure 1F). In addition, Ab27 decreased the expression of vimentin in SNU-398 and HT-29 cells (Figure 1F), indicating that Ab27 suppresses TM4SF5-induced EMT events.

Ab27 inhibits HCC growth in xenograft mouse models

In a previous study, we observed that the intratumoral injection of Ab27 decreased tumor growth in nude mice bearing TM4SF5-overexpressing SNU-449T_7 (liver cancer) subcutaneous xenografts.[14] In the present study, we investigated the antitumor activity of systemically injected Ab27 in xenograft nude mouse models. TM4SF5-overexpressing SNU-449T_7 cells ectopically expressing the luciferase gene were injected into the livers of nude mice. Starting 1 week later, Ab27 (100 μg/mouse/dose) was administered by intraperitoneal

(D) SNU-449Tp cells were treated with DyLight 488, conjugated with Ab27 (green) for 3 h at 37°C, and stained with LysoTracker red DND-99 (red). Cell nuclei were counterstained with DAPI (blue). Arrows indicate signal co-localization. Scale bar, 20 μm. (E) Cells were transfected with siRNA against *TM4SF5* for 48 h before lysis for immunoblot analysis. (F) Cells were incubated with Ab27 (250 μg/mL) for 48 h under suspension conditions before lysis for immunoblot analysis. Densitometric quantification of bands on the immunoblot was performed using GAPDH as a loading control except that phosphorylated STAT3 and FAK were normalized against the corresponding total protein (E and F). (G) Anchorage-independent growth assay in the presence of Ab27. Colonies (>0.5 mm for SNU-398 and >0.3 mm for HT-29 cells) were counted in six 100× fields per well. Values represent means ± SDs. *$p < 0.05$; **$p < 0.01$.

(i.p.) injection 3 times per week, for a total of 8 times. *In vivo* imaging analysis revealed that Ab27 inhibited tumor growth by 64% without affecting body weight (Figure 2A). Similarly, the administration of Ab27 (100 μg/mouse/dose) suppressed tumor growth by 66% when the same tumor cells were subcutaneously injected into the flanks of nude mice (Figure S2).

We also evaluated the antitumor activity of Ab27 in a sorafenib-resistant SNU-449T_7 cell xenograft model. TM4SF5-overexpressing SNU-449T_7 cells were treated with sorafenib (1–10 μM; 3 culture medium changes per week) for 3 weeks, and the surviving cells (sorafenib-resistant SNU-449T_7) were injected subcutaneously into the flanks of nude mice. As shown in Figure 2B, the administration of Ab27 (250 μg/mouse/dose) by i.p. injection (3 times per week for a total of 8 times) inhibited tumor growth by 50%, based on mean tumor volume, whereas sorafenib (400 μg/mouse/dose) did not decrease tumor growth, as expected. Immunoblot analysis of xenograft tumor tissues showed that Ab27 decreased the phosphorylation of FAK (moderately), c-Src (moderately), $p27^{Kip1}$, and STAT3, and the expression of BMI1, whereas sorafenib moderately decreased the phosphorylation of FAK, c-Src, $p27^{Kip1}$, and STAT3 and the expression of BMI1 (Figure 2C). Of note, consistent with our previous observation,[14] the expression of $p27^{Kip1}$ was not substantially changed by Ab27 in xenograft models established using SNU-449T_7 cells. This may be due to the phosphorylation of $p27^{Kip1}$ at sites other than Ser10, affecting protein stabilization.

We also investigated the antitumor activity of Ab27 in nude mice bearing liver cancer xenografts of SNU-398 cells expressing endogenous TM4SF5. Ab27 (300 μg/mouse/dose), cetuximab (300 μg/mouse/dose), or sorafenib (600 μg/mouse/dose) was i.p. injected at 2- or 3-day intervals a total of 6 times. As shown in Figure 2D, Ab27 inhibited tumor growth by 46% without affecting body weight. The antitumor activity of Ab27 was comparable with that of cetuximab and sorafenib, which inhibited tumor growth by 50% and 51%, respectively. Immunostaining of tumor sections showed that injection of Ab27, cetuximab, or sorafenib significantly decreased the Ki67 proliferation index (Figure 2E).

Antitumor activity of Ab27 against colon cancer growth in a xenograft model in mice

Previously, we observed that high levels of TM4SF5 correlated with worse overall survival of CRC patients,[14] suggesting a potential role for TM4SF5 in colon cancer progression. Therefore, we investigated the antitumor efficacy of Ab27 in nude mice bearing colon cancer xenografts of HT-29 cells expressing endogenous TM4SF5. Ab27 (142 μg/mouse/dose) was i.p. injected twice per week a total of 6 times into xenografted mice. Ab27 administration inhibited tumor growth by 38% without affecting body weight (Figure S3A). Immunostaining of tumor sections showed that the number of proliferative $Ki67^+$ cells (outside the region of cell death) was significantly lower in tumors from mice injected with Ab27 than in tumors from control mice (Figure S3B). In addition, Ab27-treated tumors exhibited a larger area of cell death within the tumor core than control tumors (Figure S3C), indicating that Ab27 can accelerate necrosis or apoptosis induced by hypoxia.[17] Consistent with this, Ab27 promoted apoptosis in HT-29 cells grown under suspension conditions *in vitro* (Figure S3D). These results indicate that Ab27 slows tumor growth *in vivo* by inhibiting tumor cell proliferation and promoting tumor cell death. Immunoblot analysis of tumor tissues revealed that Ab27 decreased the phosphorylation of $p27^{Kip1}$ and STAT3 and the expression of $p27^{Kip1}$ and BMI1, but had a less substantial effect on the phosphorylation of extracellular signal-regulated kinase 1/2 (ERK1/2) (Figure S3E). These results indicate that Ab27 inhibits tumor growth in nude mice bearing $TM4SF5^+$ liver and colon cancer xenografts and that this occurs concomitantly with a reduction in the phosphorylation levels of STAT3 and $p27^{Kip1}$.

Antitumor efficacy of combined treatment with Ab27 and sorafenib or doxorubicin

Sorafenib is a standard regimen for advanced HCC, and doxorubicin is an anticancer agent used for transcatheter arterial chemoembolization (TACE) of HCC. For combined treatment with Ab27 and sorafenib or doxorubicin, we performed preliminary experiments to evaluate the antitumor efficacies of sorafenib and doxorubicin in the SNU-449T_7 xenograft mouse model. In the next experiment, submaximal schedules or doses of Ab27, sorafenib, and doxorubicin were injected into the SNU-449T_7 xenograft model to examine whether combined treatment with Ab27 and the drug of interest would inhibit tumor growth to a greater extent than Ab27 or drug alone. Ab27 (270 μg/mouse/dose), sorafenib (1200 μg/mouse/dose), doxorubicin (20 μg/mouse/dose), a combination of Ab27 (270 μg/mouse/dose) and sorafenib (1200 μg/mouse/dose) or doxorubicin (20 μg/mouse/dose), or saline (control) was i.p. injected twice per week a total of 6 times into nude mice ($n = 6$ per group) bearing SNU-449T_7 xenografts. As shown in Figure 3A, Ab27, sorafenib, and doxorubicin single treatment inhibited tumor growth relative to the control by 25%, 42%, and 26%, respectively, whereas combined treatment with Ab27 and sorafenib inhibited tumor growth by 54%, and combined treatment with Ab27 and doxorubicin inhibited tumor growth by 52%. Combined treatments inhibited tumor growth to a significantly greater extent than single treatments. Neither combined nor single-agent treatment affected body weight (Figure 3B). Immunoblot analysis of tumor lysates revealed that, relative to single treatment, combined treatment with Ab27 and sorafenib increased the inhibition of phosphorylation of FAK and $p27^{Kip1}$ and that combined treatment with Ab27 and doxorubicin increased the inhibition of the phosphorylation of FAK, $p27^{Kip1}$, and STAT3 (Figure 3C). Notably in this regard, sorafenib abolished phosphorylation of STAT3, so it was not feasible to determine the effect of combined treatment. Reduction of BMI1 by Ab27 was confirmed, although additive reduction was not observed following combined treatment. These results indicate that combined treatment with Ab27 and sorafenib or doxorubicin inhibits tumor growth more strongly than treatment with antibody or drug alone.

In vivo toxicity of Ab27

We next examined the cross-reactivity of Ab27 with mouse TM4SF5. ELISA showed that Ab27 did not bind to recombinant mouse

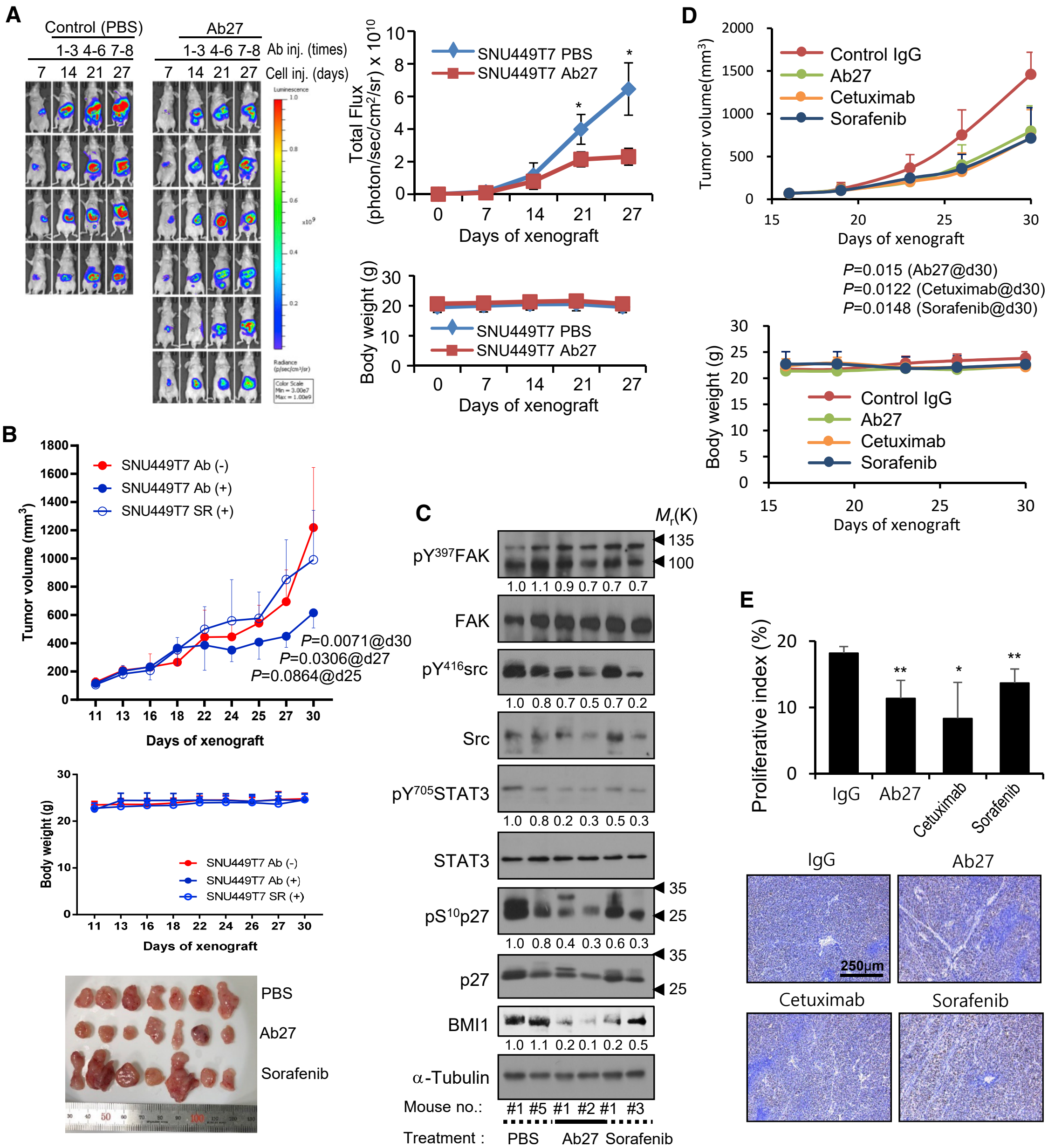

Figure 2. Ab27 inhibits HCC growth in xenograft mouse models

(A) SNU-449T7-luc (stably overexpressing TM4SF5 and luciferase) cells (5×10^5) were injected orthotopically into mouse liver after minimal incision. On day 7, Ab27 (100 μg/mouse) was i.p. injected 2 or 3 times per week for 3 weeks (total of 8 injections). PBS was injected as a negative control. Left: Up to 27 days after cell injection, bioluminescence images were acquired. Right upper: Total bioluminescence flux for 3 weeks of treatment. Right lower: Body weight of injected mice. (B and C) Sorafenib-resistant SNU-449T7 (1×10^6) cells were mixed with Matrigel and injected subcutaneously into the backs of mice. Ab27 (250 μg/mouse) or sorafenib (400 μg/mouse) was i.p. injected at 2- or 3-day intervals (total of 8 injections). (B) Top: Tumor volume (length × width^2/2). The minimum value in each group was excluded from the mean calculation. Center: Body weight of injected mice. Bottom: Photographs of dissected tumor masses on day 30. (C) Immunoblot analysis of tumor extracts. Densitometric quantification of bands on the immunoblot was performed using α-tubulin as a loading control, except for phosphorylated proteins, which were normalized against the corresponding total protein. (D and E) SNU-398 cells (1×10^7) were injected subcutaneously into the flanks of mice. Ab27 (300 μg/mouse), cetuximab (300 μg/mouse), or sorafenib (600 μg/mouse) was i.p. injected into mice (total of 6 injections). Normal human IgG (300 μg/mouse) was injected as a negative control. Top: Tumor volume (length × width^2/2). Bottom: Body weight of injected mice. (E) Ki67 staining of tumor sections was performed to measure the level of cell proliferation. Representative images are shown. Scale bar, 250 μm. Values represent means ± SDs. *$p < 0.05$; **$p < 0.01$. p value is shown on the graph (B and D).

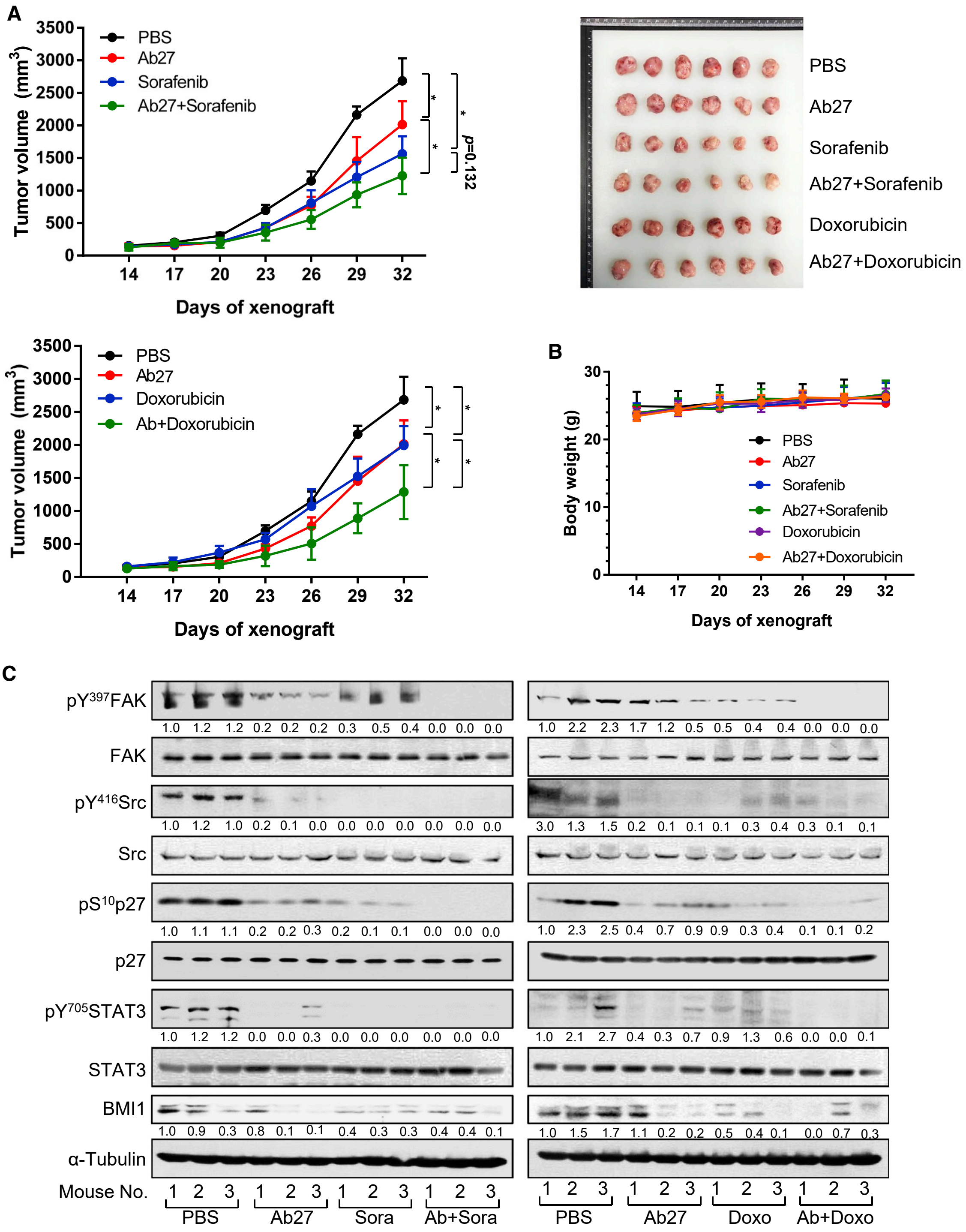

Figure 3. Antitumor efficacy of combined treatment with Ab27 and sorafenib or doxorubicin

(A) SNU-449T$_7$ (1×10^6) cells were mixed with Matrigel and then injected subcutaneously into the backs of mice. Single or combined treatment with Ab27 (270 μg/mouse), sorafenib (1,200 μg/mouse), or doxorubicin (20 μg/mouse) was performed by i.p. injection twice per week (total of 6 injections). Tumor volumes (length $\times$ width^2/2) are shown following combined treatment with Ab27 and sorafenib (left upper) or Ab27 and doxorubicin (left lower). Values for both the IgG control and Ab27 treatment groups are presented repeatedly in both graphs. Right: Photographs of dissected tumor masses on day 32 after the injection of the tumor cells. (B) Body weights of injected mice. (C) Immunoblot analysis of tumor extracts from (A). Densitometric quantification of bands on the immunoblot was performed using α-tubulin as a loading control, except for phosphorylated proteins, which were normalized against the corresponding total protein. Values represent means ± SDs. *$p < 0.05$.

TM4SF5 EC2 protein as efficiently as it did to recombinant human TM4SF5 EC2 protein (Figure S4). By contrast, flow cytometry and immunofluorescence analyses showed that Ab27 recognized TM4SF5 on CT26 mouse colon cancer cells (Figures 4A and 4B). In addition, we transiently transfected PC3 human prostate cancer cells, which normally exhibit low endogenous TM4SF5 expression, with mouse TM4SF5 expression vector. Flow cytometry revealed that Ab27 bound to PC3 cells transfected with mouse TM4SF5 expression vector more efficiently than to PC3 cells transfected with empty vector (Figure 4C), confirming reactivity with mouse TM4SF5. Therefore, the mouse is an appropriate species for studying safety *in vivo*.

To evaluate the *in vivo* toxicity of anti-TM4SF5 antibody, we administered a single dose of Ab27 (48 mg/kg) to ICR mice (female, n = 5) via intravenous (i.v.) injection and analyzed blood samples 28 days after antibody injection. *TM4SF5* mRNA is expressed in the liver (https://genecards.org), but we did not detect TM4SF5 protein in this tissue (S.K., unpublished data). Liver function was determined by measuring serum concentrations of general parameters, including alanine aminotransferase (ALT), aspartate aminotransferase (AST), alkaline phosphatase (ALP), γ-glutamyl transpeptidase (GGT), bilirubin, and albumin. We observed no general toxicities, including significant changes in liver function or body weight (Figure 4D), indicating that Ab27 is not significantly toxic *in vivo*.

Generation and characterization of humanized monoclonal antibody Ab27-hz9

For clinical applications of monoclonal antibodies, murine sequences must be humanized to decrease immunogenicity in humans.[18] Therefore, we constructed a humanized Ab27 antibody by grafting murine complementarity-determining regions (CDRs) into similar human germline sequences. Germline genes have fewer intraclonal somatic hypermutations, which can be recognized as immunogenic.[18] The human germline V genes IGHV1-2 and IGKV3-20 were selected as human acceptor frameworks for the grafting of murine CDRs. However, such CDR grafting often results in partial or complete loss of affinity of the humanized antibody. Therefore, some residues from the murine framework sequences need to be retained by replacing human residues at the corresponding positions to restore some of the lost affinity.[19] Therefore, we also engrafted murine framework sequences H71 and H73 into the human framework. The final humanized antibody was named Ab27-hz9. *In silico* immunogenicity analysis predicted that the immunogenicity of Ab27-hz9 VH would be lower than that of adalimumab and omalizumab, which are currently used in the clinic, while the immunogenicity of the Ab27-hz9 light-chain variable domain (VL) was predicted to be comparable to those of adalimumab and omalizumab (Figure S5).

We produced humanized antibody Ab27-hz9 in Expi293 cells (Figure S6) and evaluated its reactivity. The binding affinities (K_D) of Ab27 and Ab27-hz9 for human TM4SF5 (recombinant human TM4SF5 EC2-GST fusion protein) were 2.4 and 4.8 nM (Figure 5A), respectively, as measured by competitive ELISA. Next, we assessed the antigen-binding kinetics of Ab27 and Ab27-hz9 for human TM4SF5 by surface plasmon resonance (SPR) analysis. The binding affinities of Ab27 and Ab27-hz9 were 5.92 and 8.73 nM (Figure 5B), respectively, indicating that the affinity of Ab27-hz9 was 1.47-fold lower than that of Ab27. Flow cytometry of SNU-449Cp and SNU-449Tp cells revealed that Ab27-hz9 and Ab27 bound more efficiently to SNU-449Tp cells than to SNU-449Cp cells (Figure 5C). Immunofluorescence staining also showed that Ab27 and, particularly, Ab27-hz9 stained the membrane edges of SNU-449Tp cells, but not those of SNU-449Cp cells (Figure 5D). These results indicate that Ab27-hz9 recognizes TM4SF5 on the cell surface at least as strongly as Ab27. Internalization analysis by flow cytometry revealed that the residual levels of cell surface-bound Ab27-hz9 and Ab27 were substantially reduced after binding to TM4SF5 (Figure 5E), suggesting that Ab27-hz9 decreased the level of cell-surface TM4SF5 as effectively as Ab27. These findings indicate that the humanized antibody Ab27-hz9 is fully reactive to TM4SF5 protein and recognizes its target on the cell surface more efficiently than the original antibody Ab27.

In vivo antitumor activity of Ab27-hz9 in a liver cancer xenograft model

Next, we evaluated the antitumor efficacy of Ab27-hz9 in the SNU-449T_7 xenograft model. Ab27-hz9, Ab27, cetuximab, or normal human IgG (300 μg/mouse/dose) was i.p. injected 3 or 4 times per week (total 12 times) into nude mice bearing the SNU-449T_7 xenograft. Ab27-hz9 and Ab27 inhibited tumor growth by 66% and 62%, respectively, without affecting body weight (Figure 5F), which is comparable to the 59% inhibition induced by cetuximab. Immunostaining of tumor sections revealed that the level of proliferative Ki67$^+$ cells was significantly lower in tumors from mice injected with Ab27-hz9 or Ab27 than in tumors from control mice (Figure 5G), indicating that Ab27-hz9 decreased tumor growth by suppressing tumor cell proliferation as efficiently as Ab27.

In vivo tumor targeting of Ab27 and Ab27-hz9 in xenograft models

To validate the tumor-targeting ability of anti-TM4SF5 antibodies, we evaluated the distributions of Ab27 and Ab27-hz9 after injection into the SNU-449Tp xenograft model. Ab27, Ab27-hz9, and control human IgG were conjugated with a fluorescent dye (DyLight 755) and i.v. injected into nude mice bearing SNU-449Tp cell-derived tumors. After 96 h, the distribution of the dye-labeled antibody was quantified by measuring the total photon flux of the fluorescence. Ab27 and Ab27-hz9 were predominantly localized in the tumor, whereas control IgG was mostly in the liver (Figure 6). In addition, we examined *in vivo* tumor targeting of Ab27 and Ab27-hz9 using the SNU-398 xenograft model. As shown in Figure 6, both antibodies were predominantly localized to the endogenous TM4SF5-expressing tumor, whereas control IgG was mainly detected in the liver (Figure S7). These results suggest that Ab27 and Ab27-hz9 can target tumor cells expressing TM4SF5 *in vivo*.

DISCUSSION

In our previous study,[14] we evaluated the antitumor effect of intratumorally injected Ab27 in a xenograft model established using the

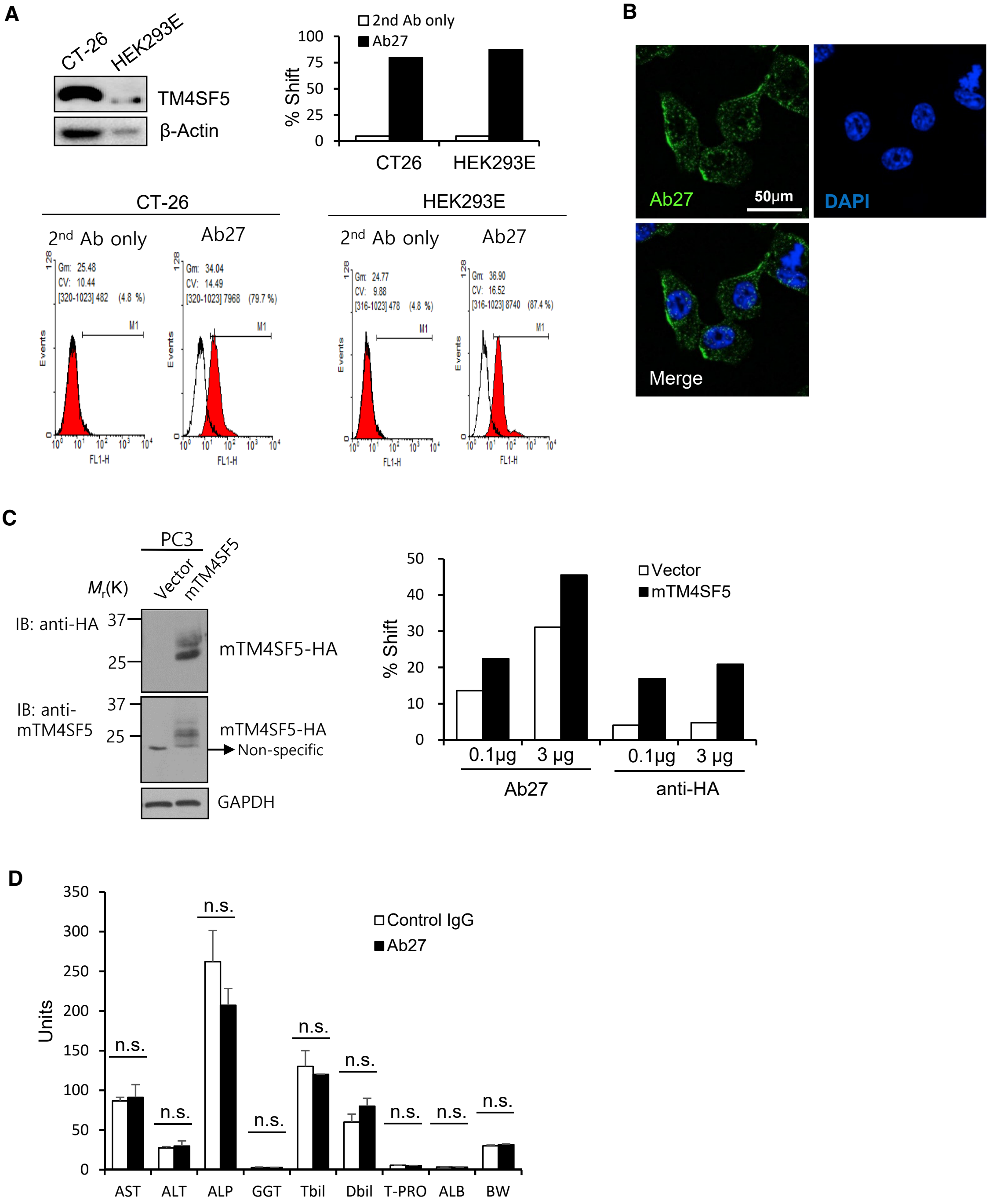

Figure 4. Cross-reactivity and *in vivo* toxicity of Ab27

(A) Immunoblot analysis with rabbit anti-TM4SF5 (in-house) and flow cytometry with Ab27 (0.05 μg/sample). (B) CT-26 cells were immunostained with Ab27 (3 μg/mL) (green). Cell nuclei were counterstained with DAPI (blue). Scale bar, 50 μm. (C) PC3 cells were transfected with HA-tagged mouse TM4SF5-expression vector for 48 h. Left, immunoblot analysis with anti-HA and anti-mouse TM4SF5 (in-house) antibodies. Right, flow cytometry with Ab27. (D) ICR mice were i.v. injected with Ab27 (48 mg/kg) or control IgG. Liver function was assessed 28 days post-injection by measuring serum concentrations of ALT, AST, ALP, GGT, Tbil, Dbil, ALB, and T-PRO. ALT, alanine aminotransferase; ALB, albumin; ALP, alkaline phosphatase; AST, aspartate aminotransferase; BW, body weight; Dbil, direct bilirubin; GGT, γ-glutamyl transpeptidase; Tbil, total bilirubin; T-PRO, total protein. Values represent means ± SDs.

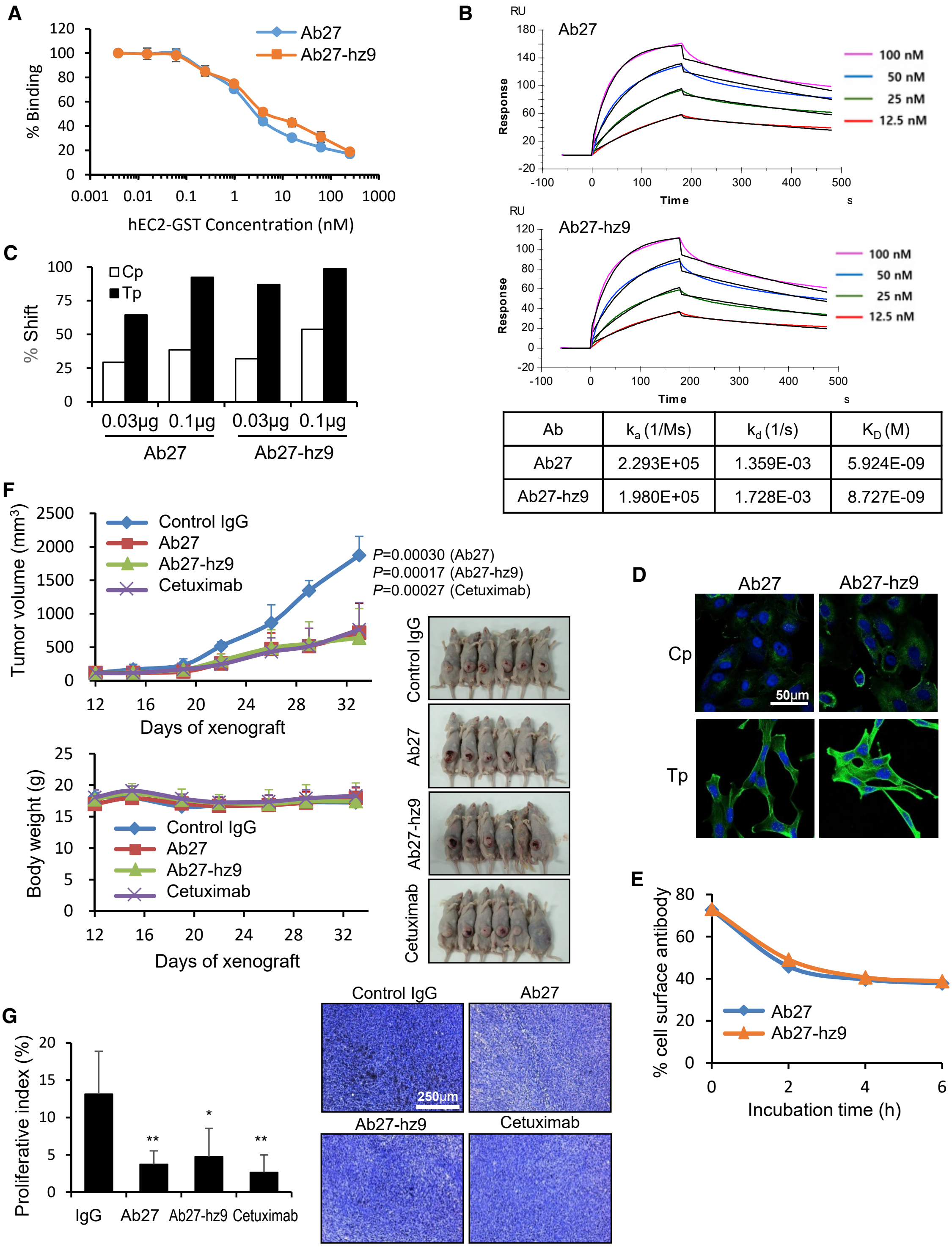

Ab	k_a (1/Ms)	k_d (1/s)	K_D (M)
Ab27	2.293E+05	1.359E-03	5.924E-09
Ab27-hz9	1.980E+05	1.728E-03	8.727E-09

Figure 5. Target recognition and antitumor activity of humanized antibody Ab27-hz9

(A and B) Affinities of Ab27 and Ab27-hz9 for recombinant human EC2-GST protein were determined using competition ELISA (A) and a BIAcore T200 system (B). k_a, association rate; k_d, dissociation rate. (C and D) Flow cytometry (C) and immunocytochemistry (D) of SNU-449Cp and SNU-449Tp cells with Ab27 and Ab27-hz9. The extent of a shift in the fluorescence signal compared to control staining, representing binding activity of antibody, is shown as a graph (C). Scale bar, 50 μm. (E) Internalization analysis of SNU-449Tp cells with Ab27 and Ab27-hz9, as described in Figure 1C. Of note, Ab27 (0.3 μg/mL) and Ab27-hz9 (0.2 μg/mL) were used to maintain a similar extent

(legend continued on next page)

stably TM4SF5-expressing liver cancer cell line SNU-449T$_7$. We observed that Ab27 significantly inhibited tumor growth, demonstrating the antitumor efficacy of Ab27. However, this study was limited by the administration method and the use of a xenograft model established using the TM4SF5-overexpressing stable cancer cell line. To improve the clinical potential of this antibody, in this study, we evaluated the therapeutic potential of Ab27 using endogenous or stably TM4SF5-expressing liver and colon cancer cells *in vivo* and *in vitro*. Our results demonstrate that systemic administration of Ab27 significantly suppressed tumor growth in both endogenous and stably TM4SF5-expressing HCC and colon cancer xenograft models. In particular, Ab27 efficiently reduced tumor growth in a sorafenib-resistant model. Combination treatment with Ab27 and sorafenib or doxorubicin increased antitumor activity relative to monotherapy. Ab27 did not exert general toxicity or interfere with normal liver function *in vivo*. Consistent with this, TM4SF5-KO (*Tm4sf5*$^{-/-}$) mice generated in our previous study[20] did not exhibit any abnormal phenotypes at ages up to 24 months (J.W.L., unpublished data). These results suggest that TM4SF5 has potential as a therapeutic target for the treatment of HCC and CRC, and that it is likely that therapeutic blocking of TM4SF5 is safe.

In addition, the humanization process via CDR grafting and back-mutation of Ab27 successfully generated Ab27-hz9, which exhibited immunogenicity lower than or comparable to those of antibodies used in the clinic while retaining reactivity to TM4SF5 protein. Therefore, Ab27-hz9 could serve as a platform for the further development of clinical treatments for liver cancer and CRC. We are generating improved antibodies by performing affinity maturation of Ab27-hz9 through mutation of the CDR sequences after conversion of the antibody to a whole-IgG format. The final optimized antibodies will be reported upon completion.

Sorafenib, a multi-kinase inhibitor that inhibits Raf-1, B-Raf, VEGF receptors, and platelet growth factor receptor, is the first-line systemic therapy that has demonstrated a significant increase in mean overall survival in patients with advanced HCC. However, its clinical benefit is modest due to the high resistance rate.[2] Potential mechanisms responsible for the acquisition of sorafenib resistance include EMT, the presence of cancer stem cells, EGFR activation, and autophagy.[3,21] In our previous studies, we showed that TM4SF5 mediates EMT, cell migration, tumorigenesis, and drug resistance by associating with integrins $\alpha2$, $\alpha5$, and $\beta1$, as well as EGFR.[9,11] TM4SF5-mediated EMT may have an important function in the resistance of lung cancer cells to EGFR kinase inhibitors (e.g., gefitinib).[11] The anti-TM4SF5 compound TSAHC also inhibits TM4SF5-mediated tumor growth in a manner that is distinct from the antitumor effect of sorafenib.[22] Therefore, blocking TM4SF5 may have the potential to overcome sorafenib resistance. In this study, we observed that Ab27 exhibited antitumor activity comparable with that of sorafenib and decreased tumor growth in a sorafenib-resistant HCC xenograft model. In addition, combination treatment with Ab27 and sorafenib or doxorubicin exerted higher antitumor activity than either drug alone, suggesting the potential benefit of combination treatments in systemic injection or TACE treatment. Sorafenib inhibits the phosphorylation of STAT3 at Tyr705, thereby suppressing growth and inducing apoptosis in HCC,[23] indicating that STAT3 contributes to the antitumor effects of sorafenib on HCC. In addition, sorafenib-mediated upregulation of STAT3 in HCC cells contributes to sorafenib resistance,[24] and combination treatment with a STAT3 inhibitor and sorafenib synergistically increases the antitumor effect of sorafenib on HCC cells.[25] Thus, STAT3 is not only an indirect target of sorafenib but it also mediates sorafenib resistance. In a previous study, we showed that TM4SF5 activates STAT3 signaling to promote the spheroid formation and self-renewal of HCC.[10] Here, we showed that an anti-TM4SF5 antibody suppressed STAT3 phosphorylation, contributing to antitumor activity in a sorafenib-resistant model and increasing antitumor activity in combination with sorafenib. Therefore, blocking TM4SF5 is a candidate strategy against HCC cells resistant to sorafenib.

EGFR is a receptor tyrosine kinase involved in multiple cancers. EGFR-inhibiting agents such as EGFR-blocking antibodies or EGFR tyrosine kinase inhibitors have been approved to treat various cancers, including colorectal and non-small cell lung cancers.[26] In addition, blocking EGFR could be used to overcome the sorafenib resistance of HCC.[3] TM4SF5 is aberrantly expressed in liver, colon, lung, pancreatic, and esophageal cancers, and its high expression is associated with a poor prognosis.[8,27] We observed that Ab27 and Ab27-hz9 suppressed *in vivo* tumor growth to the same extent as cetuximab. The anti-TM4SF5 strategy may have therapeutic advantages for the treatment of metastatic CRC and HCC because it targets different pathways from the current therapeutics, and Ab27-hz9 could be exploited as a starting platform to establish anti-TM4SF5 strategy. Further evaluation of Ab27-hz9 in CRC, lung, and pancreatic cancer models, alone or in combination with current therapeutics, would be worthwhile.

Antibody-drug conjugates (ADCs) are monoclonal antibodies conjugated to highly cytotoxic small molecules through chemical linkers and have emerged as a potential strategy for the treatment of several cancers. ADCs enable the targeting of cancer cells and selective delivery of potent cytotoxic drugs.[28] To develop an ADC, the antibody must target a cell-surface receptor that is highly expressed on the targeted cancer cells; in addition, it must be internalized to deliver the drug inside the cells.[28] We observed that Ab27 and Ab27-hz9 bound to cell-surface TM4SF5 and were internalized in tumor cells. In addition, these antibodies efficiently targeted TM4SF5$^+$ tumors *in vivo*.

of initial antibody binding to TM4SF5 on the cell surface. (F and G) SNU-449T$_7$ cells (3×10^6) were subcutaneously injected into the flanks of mice. Normal human IgG (negative control), Ab27, Ab27-hz9, or cetuximab (300 μg/mouse) was i.p. injected (total of 12 injections). (F) Top: Tumor volume (length × width^2/2). Bottom: Body weight of injected mice. Right: Photographs of tumor-bearing mice on day 33. (G) Ki67 staining of tumor sections. Scale bar, 250 μm. Values represent means ± SDs. *$p < 0.05$; **$p < 0.01$. p value is shown on the graph (F).

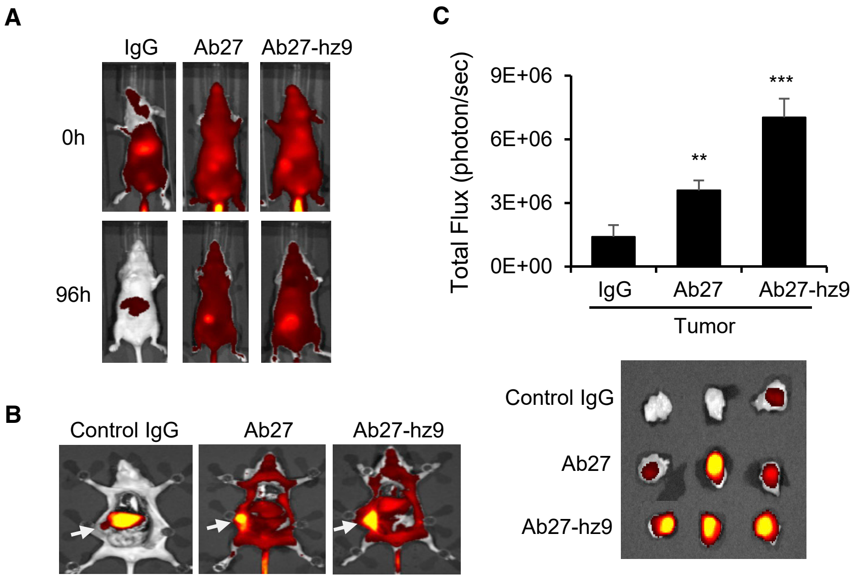

Figure 6. *In vivo* tumor targeting of Ab27 and Ab27-hz9 in a xenograft model
SNU-449T$_7$ cells were subcutaneously injected into the flanks of nude mice to generate tumor-bearing mice. DyLight 755-labeled Ab27, Ab27-hz9, or normal human IgG was injected into the tail vein of tumor-bearing mice, and fluorescence was measured after 96 h. (A) Whole body of mice. (B) Dissected mice at 96 h. Arrow indicates tumor mass. (C) Total fluorescence flux of tumor mass was measured (top). Dissected tumor mass (bottom). Values represent means ± SDs. $^{**}p < 0.01$; $^{***}p < 0.001$.

Based on the internalization and lysosomal targeting of Ab27, we generated an ADC using Ab27 and a DNA crosslinking agent. The Ab27-based ADC exhibited dramatic antitumor activity *in vitro* and in a colon cancer xenograft model (S.K., unpublished data), suggesting that Ab27-based ADCs could serve as potent therapeutics for the treatment of TM4SF5$^+$ cancers. In addition, immune cell therapy with adoptively transferred chimeric antigen receptor (CAR)-T cells is being actively investigated for the treatment of solid tumors after the reported success of CD19-targeting CAR-T cell therapy against B cell malignancies.[29,30] Thus, it would be interesting to generate and evaluate the effects of anti-TM4SF5 CAR-T cells based on the Ab27-hz9 scFv sequence in HCC and CRC models.

In summary, we evaluated the therapeutic potential of the anti-TM4SF5 chimeric antibody Ab27 using liver and colon cancer cells expressing TM4SF5 *in vitro* and *in vivo*. Ab27 suppressed tumor growth in liver and colon cancer xenograft models, including sorafenib-resistant HCC, which occurred concomitantly with a reduction in the phosphorylation levels of FAK, p27^{Kip1}, and STAT3. Combination treatment with Ab27 and sorafenib or doxorubicin exerted higher antitumor activity than monotherapy. No general toxicities of Ab27 were observed *in vivo*. Furthermore, we developed a humanized antibody Ab27-hz9 via the CDR grafting technique. Ab27-hz9 exhibited specific target recognition and antitumor activity comparable with those of Ab27, indicating that Ab27-hz9 could serve as a starting platform for further development of clinical treatments for liver cancer and CRC.

MATERIALS AND METHODS

Production of scFv-hFc

The anti-TM4SF5 chimeric monoclonal antibody Ab27 and its humanized monoclonal antibody Ab27-hz9 were produced using the Expi293 or the ExpiCHO Expression system (Thermo Fisher Scientific, Waltham, MA, USA) and purified using protein A affinity chromatography with MabSelect SuRe Column (GE Healthcare, Uppsala, Sweden) or as previously described.[14]

Cell cultures

Human embryonic kidney 293E (HEK293E), HCT-116, HT-29 (colon cancer), HepG2 (liver cancer), PC3 (prostate cancer), and CT26 (mouse colon cancer) cell lines were purchased from the American Type Culture Collection (ATCC; Manassas, VA, USA). SNU-398 (liver cancer) and SNU-638 (gastric cancer) cell lines were purchased from the Korean Cell Line Bank (KCLB; Seoul, Korea). HEK293E and HepG2 cells were maintained in DMEM with 10% fetal bovine serum (FBS) at 37°C in 5% CO_2. HCT-116, HT-29, PC3, SNU-398, SNU-638, and CT26 cells were maintained in RPMI1640 with 10% FBS at 37°C in 5% CO_2. The stable SNU-449Cp (TM4SF5-low), SNU-449Tp, and SNU-449T$_7$ (both highly TM4SF5$^+$) liver cancer transfectant cell lines were maintained as previously described.[7] Luciferase-expressing SNU-449T$_7$ cells (SNU-449T$_7$-luc) were previously described.[10] C8161 cells (melanoma) were a kind gift from Dr. C.-R. Jung (Korea Research Institute of Bioscience and Biotechnology [KRIBB], Daejeon, Korea).[31]

Transfection with siRNA

Cells were transfected with siRNA specific to *TM4SF5* (5′-GGACCAACACCAACCATCTCAGCTT-3′) and scrambled siRNA (5′-ACGCACCACGATCTATATCGCCAAC-3′) using lipofectamine 2000 for 48 h before analysis. *STAT3*-specific siRNA (5′-CAGCCTCTCTGCAGAATTCAA-3′) was also used.

Flow cytometry

To analyze antibody binding to TM4SF5, flow cytometry was performed using SNU-449Cp, SNU-449Tp, HEK293E, and CT26 cells. HCT-116, SNU-398, HT-29, C8161, and SNU-638 cells that had been transiently transfected with either a *TM4SF5*-specific siRNA or a negative control siRNA were also analyzed. Cells (2×10^5) were incubated with Ab27 or Ab27-hz9 at 0.03–0.3 μg for 45 min at 4°C in 100 μL PBS containing 1% BSA. The cells were washed twice with 1% BSA/PBS, followed by a 30-min incubation with fluorescein isothiocyanate (FITC)-conjugated anti-human IgG (Fc-specific; Pierce,

Rockford, IL, USA). Viable propidium iodide $(PI)^-$ cells were analyzed for antibody binding using a FACSCalibur (BD Immunocytometry System, San Jose, CA, USA). To generate hemagglutinin (HA)-tagged mouse TM4SF5-expression vector, mouse *Tm4sf5* cDNA (GenBank: NM_029360.3) was subcloned into pCMV-HA-N vector (Clontech, Mountain View, CA, USA). PC3 cells transiently transfected with HA-tagged mouse TM4SF5-expression vector were incubated with Ab27 or anti-HA, and then analyzed by flow cytometry.

Immunoblot analysis

Whole-cell lysates were prepared using radioimmunoprecipitation assay (RIPA) buffer, immunoblotted as described,[32,33] and analyzed using the following primary antibodies: anti-FAK, anti-phospho-p27 (S10), anti-p27, anti-c-Src, anti-β-actin, anti-α-tubulin, and anti-GAPDH (glyceraldehyde 3-phosphate dehydrogenase; Santa Cruz Biotechnology, Santa Cruz, CA, USA); anti-phospho-c-Src (Y416), anti-phospho-ERK1/2, anti-ERK1/2, anti-phospho-STAT3 (Y705), and anti-STAT3 (Cell Signaling, Danvers, MA, USA); anti-phospho-FAK (Y397) (Abcam, Cambridge, UK); anti-BMI1 (Millipore, Temecula, CA); anti-vimentin (Sigma, St. Louis, MO); anti-HA (Roche, Mannheim, Germany); and rabbit anti-human TM4SF5[7] and rabbit anti-mouse TM4SF5, which was produced using a peptide-corresponding mouse TM4SF5 (amino acid residues 117–138; CLIDNKWDYHFQETEGAYLRND) by ProSci (Poway, CA, USA).

Immunocytochemistry

SNU-449Cp, SNU-449Tp, and HepG2 and HT-29 cells that had been transiently transfected with *TM4SF5*-specific siRNA were plated on coverslips and incubated for 48 h. The cells were then fixed for 20 min in methanol and permeabilized for 1 min with acetone. After blocking in 1% normal horse serum, the cells were incubated with Ab27 or Ab27-hz9 (3 or 5 μg/mL), followed by a corresponding secondary antibody conjugated to FITC. The cells were counterstained with 4,6-diamidino-2-phenylindole (DAPI; Sigma) to visualize nuclei. Immunofluorescent images were acquired under a confocal microscope (LSM 510 META; Carl Zeiss, Jena, Germany).

Internalization analysis

Cell-surface binding and subsequent internalization of Ab27 and Ab27-hz9 were analyzed using flow cytometry. Briefly, cells were incubated with Ab27 or Ab27-hz9 for 45 min at 4°C, washed to remove unbound antibodies, and then either warmed to 37°C to allow internalization or maintained at 4°C. Cells were transferred to ice-cold buffer to stop the occurrence of internalization and stained with FITC-conjugated anti-human IgG. PI^- cells were analyzed by flow cytometry. The relative levels of Ab27 or Ab27-hz9 bound to the cell surface were calculated as the shift in the fluorescence signal of the antibody occupancy relative to that detected at the beginning of the internalization period.

To detect lysosomal localization of Ab27, SNU-449Tp cells were seeded on coverslips in a 6-well plate and incubated for 48 h at 37°C. Cells were incubated with Ab27 conjugated to DyLight 755 for 3 h at 37°C and stained with 200 nM LysoTracker red DND-99 (Thermo Fisher Scientific) for 2 h at 37°C. The cells were counterstained with DAPI before observation with a confocal microscope.

ELISAs (antigen-binding and competition ELISAs)

The recombinant human EC2-Fc fusion protein (EC2 [amino acid residues 113–157] fused to the Fc of human IgG1) was previously described.[14] Recombinant human EC2-mouse Fc (derived from mouse IgG2a) fusion protein and mouse EC2 (amino acid residues 112–156)-mouse Fc fusion protein were produced in a similar way. A DNA fragment encoding human EC2 followed by glutathione S-transferase (GST) was subcloned into the *pET21* vector (Novagen, Darmstadt, Germany). The recombinant human EC2 domain fused with GST was expressed in *E. coli* BL21 (DE3) and affinity purified using a glutathioneSepharose 4B column (GE Healthcare).

Ab27 scFv sequence was subcloned into the pComb3X vector digested with SfiI. Ab27 scFv tagged with 6×His and HA was expressed in *Escherichia coli* BL21 by isopropyl β-D-1-thiogalactopyranoside (IPTG) induction (final concentration of 0.1 mM), and a periplasmic extract was obtained using the osmotic shock method.[34] Ab27 of the scFv-6×His-HA format was purified from the periplasmic extract by Ni-NTA affinity chromatography.

For antigen-binding ELISA, 96-well immunoplates (eBiosciences, San Diego, CA, USA) were coated with the purified hEC2-GST protein (100 ng/well) diluted in 50 mM sodium carbonate buffer (pH 9.6) at 4°C overnight and then blocked with 2% BSA in PBS. The plates were washed 3 times with PBS containing 0.05% Tween 20 between all of the steps. Ab27 or Ab27-hz9 (amounts ranging from 0 to 90 ng/well) was added into each well, and then horseradish peroxidase (HRP)-conjugated anti-human Fc was added. All of the incubations were carried out at 37°C for 1 to 2 h. Color was developed with 3,3′,5,5′-tetramethylbenzidine (TMB) substrate solution, and the absorbance was measured at 450 nm using a microplate reader (BMG LABTECH GmbH, Ortenber, Germany). Binding of Ab27 scFv-6×His-HA to hEC2-mFc or mEC2-mFc protein was also analyzed using HRP-conjugated anti-HA in a similar manner.

For competition ELISA to determine affinity (K_D value), 96-well plates were coated with the hEC2-GST protein. Ab27 or Ab27-hz9 (amount reaching absorbance 1, which was determined from the above antigen-binding ELISA) were incubated with hEC2-GST protein ranging from 0 to 1 μM for 2 h and then the mixtures were put onto the plates for 1 h. HRP-conjugated anti-human Fc incubation, color development, and absorbance measurement were performed as described above.

Construction of humanized antibody Ab27-hz9

Six CDRs determined by combined Kabat/IMGT/Paratome numbering[35] and some framework residues (H71 and H73) of the Ab27 were grafted into the human IGHV1-2 and IGKV3-20 frameworks. Humanized heavy-chain variable domain (VH) and kappa chain variable domain (Vκ)-encoding genes were synthesized as an

scFv format and then inserted into the *pDR-OriP-Fc1* mammalian expression vector as described previously.[14]

SPR analysis

The kinetic parameters of the interaction between the scFv-Fc forms of Ab27 and Ab27-hz9 and hEC2-GST were determined at 25°C using the Biacore T200 (Cytiva, Marlborough, MA, USA). Briefly, anti-human Fc antibody was aminecoupled on a CM5 sensor chip using the Human Antibody Capture Kit (Cytiva) and then scFv-Fc was injected for 30 s at a flow rate of 10 μL/min according to the manufacturer's instructions. hEC2-GST in HBS-EP buffer with 350 mM NaCl at a concentration range of 12.5–100 nM was then injected over 3 min at a flow rate of 30 μL/min. After each binding cycle, a regeneration solution (3 M $MgCl_2$) was injected for 1 min to remove any non-covalently bound protein. The association rate (k_a), dissociation rate (k_d), and equilibrium dissociation constant (K_D, k_d/k_a) were determined using a 1:1 binding model and Biacore BIAevaluation software version 1.0.

Cell proliferation assay

Cell proliferation was determined using the colorimetric WST-1 Cell Proliferation Assay Kit (Takara Bio, Otsu, Shiga, Japan). Briefly, cells were seeded into 96-well plates at a density of 5×10^3 cells/well and incubated for 48 to 72 h in the presence of antibodies. The cells were then incubated with the WST-1 reagent (1/10th of the medium volume), and formazan dye formation was determined by measuring absorbance at 450 nm using a microplate reader.

Soft agar anchorage-independent growth assay

Cells were seeded at a density of 1×10^3 cells/well in 6-well tissue culture plates in 0.4% agar (Sigma) over a 0.6% agar feed layer. Cells were allowed to grow at 37°C in 5% CO_2 for 13 days, and the number of resulting colonies with diameter >0.3 or 0.5 mm was counted per well.

Anoikis assay

Cells (5×10^5) were seeded in the presence of antibodies into 6-well plates with an Ultra-Low Attachment Surface (Corning, Corning, NY, USA) to induce anoikis. Cells were washed and stained with 5 μL of annexin V and 5 μL of PI per 1×10^5 cells for 15 min at room temperature (RT) in the dark, and the percentage of apoptotic cells was analyzed using flow cytometry. The cells were harvested after the induction of anoikis, washed with PBS, and lysed for immunoblot analysis. HA6, a scFv-Fc recognizing the hepatitis A virus,[14] was used as a negative control antibody.

Mouse xenograft models

All of the animal procedures were performed in accordance with the procedures of the Seoul National University Laboratory Animal Maintenance Manual and Institutional Review Board (IRB) agreement (SNU-190122-6-3 and SNU-161222-3), and the guidelines of the Animal Care Committee at the Korea Research Institute of Bioscience and Biotechnology (KRIBB) and approval of the bioethics committee of KRIBB (KRIBB-AEC-17041, KRIBB-AEC-18094, KRIBB-AEC-19098, and KRIBB-AEC-20117). Nude mice (BALB/c-nude, 5 weeks old) were obtained from Japan SLC (Nishi-ku, Hamamatsu, Shizuoka, Japan) or Nara Biotech (Seoul, Korea). SNU-449T_7-luc (stably overexpressing TM4SF5 and luciferase) cells[10] were injected orthotopically into the liver (5×10^5 cells) after minimal incision or subcutaneously into the back (1×10^6 cells) of mice. On day 7 or 14, the mice were randomized into control and treatment groups. Ab27 (100 μg/mouse) was i.p. injected at 2- or 3-day intervals for 2 or 3 weeks (total 7 or 8 times). Bioluminescence from SNU-449T7 cells were acquired in an IVIS Luminar imaging system (PerkinElmer, Santa Clara, CA, USA), as previously described.[10]

SNU-449T_7 cells were treated with sorafenib (1–10 μM) for 2 weeks, and the surviving cells (sorafenib-resistant SNU-449T_7; 1×10^6 cells) were mixed with Matrigel on ice and then injected subcutaneously into the backs of mice. After 11 days, when tumor volumes reached ~100 mm^3, the tumor-bearing mice were randomized into control and treatment groups (n = 7 per group). Ab27 (250 μg/mouse) or sorafenib (400 μg/mouse) was i.p. injected at 2- or 3-day intervals (total 8 times).

SNU-398 (1×10^7) and HT-29 (2.5×10^6) cells were injected subcutaneously into the flank of each mouse. When volumes reached ~70 mm^3, the tumor-bearing mice were randomized into control and treatment groups (n = 6 per group). Ab27 (300 μg/mouse), cetuximab (300 μg/mouse), or sorafenib (600 μg/mouse) was i.p. injected into mice at a 2-day interval or twice per week (total 6 times). Normal human IgG (Sigma; 300 μg/mouse) or PBS was injected as a negative control.

For combination treatment, SNU-449T_7 (1×10^6) cells were subcutaneously injected into mice. When volumes reached ~100 mm^3, the tumor-bearing mice were randomized into control and treatment groups (n = 6 per group). Ab27 (270 μg/mouse), sorafenib (1200 μg/mouse), or doxorubicin (20 μg/mouse) alone were i.p. injected into mice twice per week (total 6 times). For the combination treatment group, Ab27 and sorafenib or doxorubicin were i.p. injected at 6-h intervals. Sorafenib was dissolved in DMSO/Cremophor EL/PBS (1:1:8).

SNU-449T_7 (3×10^6) cells were subcutaneously injected into the flanks of mice. When volumes reached ~100 mm^3, the tumor-bearing mice were randomized into control and treatment groups (n = 6 per group). Ab27, Ab27-hz9, or cetuximab (300 μg/mouse) was i.p. injected at 2- or 3-day intervals (total 12 times). Normal human IgG was injected as a negative control. Body weight and tumor volume were measured before injection of the antibody or drug. Tumor volumes were calculated using the formula $width^2 \times length/2$. Tumor masses were lysed as previously described[7] for immunoblot analysis, or tumor masses were fixed in 10% formalin and embedded in paraffin.

Immunohistochemistry

Formalin-fixed and paraffin-embedded 6-μm-thick tissue sections from xenograft tumors were processed for immunohistochemistry

analysis as per the standard protocol. Sections were stained with anti-Ki67 antibody (SP6; Abcam) using the peroxidase technique. The proliferative index (%) was determined by calculating the number of Ki67$^+$ cells relative to the total number of cells, which consisted of at least 1,000 cells per field. Five randomly selected fields from tumor sections per mouse were analyzed using ImageJ software.

In vivo toxicity of Ab27

In vivo toxicity testing of Ab27 was performed by KBIOhealth (Osong, Korea). Six-week-old ICR mice (female, n = 5) were i.v. injected with 48 mg/kg of Ab27. General clinical signs, including body weight, were monitored. Twenty-eight days after injection, the mice were sacrificed, and urine and blood samples were collected. Standard hematological analysis and urinalysis were performed. Blood samples were centrifuged at 1,700 $\times$ *g* for 10 min, and biochemical parameters of serum were analyzed using a Konelab 60i (Thermo Fisher Scientific, Vantaa, Finland).

In vivo tumor targeting

Ab27, Ab27-hz9, and normal human IgG were conjugated with DyLight 755 and purified using a DyLight 755 Antibody Labeling Kit (Thermo Fisher Scientific). Seventy micrograms of dye-labeled antibody were injected into the tail vein of nude mice bearing SNU-449T$_7$ or SNU-398 cell-derived tumors at a tumor size of $\sim$100 mm^3. The distribution profiles of the antibody were quantified by *in vivo* fluorescence using an *in vivo* imaging system (IVIS) at 0, 24, 48, 72, and 96 h after antibody injection. The tumor was removed at 96 h after injection to determine the distribution of the DyLight755-labeled antibody in tumor tissues.

Analysis of TCGA data

cBioPortal (www.cbioportal.org)[36,37] was used to analyze TCGA-generated human liver HCC data (TCGA, PanCancer Atlas). All of the patient samples with available mRNA and protein expression profiles were included in the correlation analysis.

Statistical analysis

For the statistical analysis, all of the data points were tested for normality using either the D'Agostino-Pearson or Shapiro-Wilk normality test. The Student's t test or one-way ANOVA was performed for those data points that passed normality with Dunnett's multiple comparison post-test. The Mann-Whitney test or the Kruskal-Wallis test was performed for those data points that did not pass normality. All of the statistical calculations were done using Prism software (GraphPad, San Diego, CA, USA). Pearson's test was performed for correlation analysis. $p < 0.05$ was considered statistically significant.

SUPPLEMENTAL INFORMATION

Supplemental information can be found online at https://doi.org/10.1016/j.omto.2022.01.006.

ACKNOWLEDGMENTS

We thank H. Ahn for experimental assistance. This study was supported by grants from the National Research Foundation of Korea (NRF-2018M3A9C8020027 and NRF-2016M3A9A8915731, to S.K., and NRF-2020R1A2C3008993 and NRF-2021M3A9D3024752, to J.W.L.) and the KRIBB Research Initiative Program, Republic of Korea.

AUTHOR CONTRIBUTIONS

D.K. designed and performed the experiments, analyzed and interpreted the data, and wrote the manuscript; D.K., E.K., E.-A.S., S.H.N., and J.Y. performed the *in vivo* experiments and analyzed the data; J.-S.L. and Y.L. helped perform the *in vitro* experiments and analyzed the data; S.P. and S.-Y.C. performed the antibody humanization and wrote the manuscript; K.H. performed *in silico* the immunogenicity analysis and wrote the manuscript; J.W.L. designed and supervised the *in vivo* experiments, interpreted the data, and wrote the manuscript; S.K. designed and supervised the study, interpreted the data, and wrote and revised the manuscript; and all of the authors read and approved the final manuscript.

DECLARATION OF INTERESTS

The authors declare no competing interests.

REFERENCES

1.. Ferlay, J., Colombet, M., Soerjomataram, I., Mathers, C., Parkin, D.M., Pineros, M., Znaor, A., and Bray, F. (2019). Estimating the globalcancer incidence and mortality in 2018: GLOBOCAN sources and methods. Int. J. Cancer *144*, 1941–1953.

2.. Bruix, J., Han, K.H., Gores, G., Llovet, J.M., and Mazzaferro, V. (2015). Liver cancer: approaching a personalized care. J. Hepatol. *62*, S144–S156.

3.. Niu, L., Liu, L., Yang, S., Ren, J., Lai, P.B.S., and Chen, G.G. (2017). New insights into sorafenib resistance in hepatocellular carcinoma: responsible mechanisms and promising strategies. Biochim. Biophys. Acta Rev. Cancer *1868*, 564–570.

4.. Xie, Y.H., Chen, Y.X., and Fang, J.Y. (2020). Comprehensive review of targeted therapy for colorectal cancer. Signal Transduct. Target. Ther. *5*, 22.

5.. Sala-Valdes, M., Ailane, N., Greco, C., Rubinstein, E., and Boucheix, C. (2012). Targeting tetraspanins in cancer. Expert Opin. Ther. Targets *16*, 985–997.

6.. Muller-Pillasch, F., Wallrapp, C., Lacher, U., Friess, H., Buchler, M., Adler, G., and Gress, T.M. (1998). Identification of a new tumour-associated antigen TM4SF5 and its expression in human cancer. Gene *208*, 25–30.

7.. Lee, S.A., Lee, S.Y., Cho, I.H., Oh, M.A., Kang, E.S., Kim, Y.B., Seo, W.D., Choi, S., Nam, J.O., Tamamori-Adachi, M., et al. (2008). Tetraspanin TM4SF5 mediates loss of contact inhibition through epithelial-mesenchymal transition in human hepatocarcinoma. J. Clin. Invest. *118*, 1354–1366.

8.. Wu, Y.B., Huang, Y.S., Xu, Y.P., Sun, Y.F., Yu, D.L., Zhang, X.Q., Long, X., Zhu, S.Q., Zhou, J.L., and Xu, J.J. (2013). A high level of TM4SF5 is associated with human esophageal cancer progression and poor patient survival. Dig. Dis. Sci. *58*, 2623–2633.

9.. Choi, S., Lee, S.A., Kwak, T.K., Kim, H.J., Lee, M.J., Ye, S.K., Kim, S.H., Kim, S., and Lee, J.W. (2009). Cooperation between integrin alpha5 and tetraspan TM4SF5 regulates VEGF-mediated angiogenic activity. Blood *113*, 1845–1855.

10.. Lee, D., Na, J., Ryu, J., Kim, H.J., Nam, S.H., Kang, M., Jung, J.W., Lee, M.S., Song, H.E., Choi, J., et al. (2015). Interaction of tetraspan(in) TM4SF5 with CD44 promotes self-renewal and circulating capacities of hepatocarcinoma cells. Hepatology *61*, 1978–1997.

11.. Lee, M.S., Kim, H.P., Kim, T.Y., and Lee, J.W. (2012). Gefitinib resistance of cancer cells correlated with TM4SF5-mediated epithelial-mesenchymal transition. Biochim. Biophys. Acta *1823*, 514–523.

12.. Lee, S.A., Kim, Y.M., Kwak, T.K., Kim, H.J., Kim, S., Ko, W., Kim, S.H., Park, K.H., Kim, H.J., Cho, M., et al. (2009). The extracellular loop 2 of TM4SF5 inhibits integrin alpha2 on hepatocytes under collagen type I environment. Carcinogenesis *30*, 1872–1879.

13.. Lee, S.A., Ryu, H.W., Kim, Y.M., Choi, S., Lee, M.J., Kwak, T.K., Kim, H.J., Cho, M., Park, K.H., and Lee, J.W. (2009). Blockade of four-transmembrane L6 family member 5 (TM4SF5)-mediated tumorigenicity in hepatocytes by a synthetic chalcone derivative. Hepatology *49*, 1316–1325.

14.. Ahn, H.M., Ryu, J., Song, J.M., Lee, Y., Kim, H.J., Ko, D., Choi, I., Kim, S.J., Lee, J.W., and Kim, S. (2017). Anti-cancer activity of novel TM4SF5-targeting antibodies through TM4SF5 neutralization and immune cell-mediated cytotoxicity. Theranostics *7*, 594–613.

15.. Holliger, P., and Hudson, P.J. (2005). Engineered antibody fragments and the rise of single domains. Nat. Biotechnol. *23*, 1126–1136.

16.. Subramaniam, A., Shanmugam, M.K., Perumal, E., Li, F., Nachiyappan, A., Dai, X., Swamy, S.N., Ahn, K.S., Kumar, A.P., Tan, B.K., et al. (2013). Potential role of signal transducer and activator of transcription (STAT)3 signaling pathway in inflammation, survival, proliferation and invasion of hepatocellular carcinoma. Biochim. Biophys. Acta *1835*, 46–60.

17.. Shimizu, S., Eguchi, Y., Kamiike, W., Itoh, Y., Hasegawa, J., Yamabe, K., Otsuki, Y., Matsuda, H., and Tsujimoto, Y. (1996). Induction of apoptosis as well as necrosis by hypoxia and predominant prevention of apoptosis by Bcl-2 and Bcl-XL. Cancer Res. *56*, 2161–2166.

18.. Safdari, Y., Farajnia, S., Asgharzadeh, M., and Khalili, M. (2013). Antibody humanization methods - a review and update. Biotechnol. Genet. Eng. Rev. *29*, 175–186.

19.. Ahmadzadeh, V., Farajnia, S., Feizi, M.A., and Nejad, R.A. (2014). Antibody humanization methods for development of therapeutic applications. Monoclon. Antib. Immunodiagn. Immunother. *33*, 67–73.

20.. Ryu, J., Kim, E., Kang, M.K., Song, D.G., Shin, E.A., Lee, H., Jung, J.W., Nam, S.H., Kim, J.E., Kim, H.J., et al. (2021). Differential TM4SF5-mediated SIRT1 modulation and metabolic signaling in nonalcoholic steatohepatitis progression. J. Pathol. *253*, 55–67.

21.. Xia, S., Pan, Y., Liang, Y., Xu, J., and Cai, X. (2020). The microenvironmental and metabolic aspects of sorafenib resistance in hepatocellular carcinoma. EBioMedicine *51*, 102610.

22.. Lee, S.A., Lee, M.S., Ryu, H.W., Kwak, T.K., Kim, H., Kang, M., Jung, O., Kim, H.J., Park, K.H., and Lee, J.W. (2011). Differential inhibition of transmembrane 4 L six family member 5 (TM4SF5)-mediated tumorigenesis by TSAHC and sorafenib. Cancer Biol. Ther. *11*, 330–336.

23.. Tai, W.T., Cheng, A.L., Shiau, C.W., Huang, H.P., Huang, J.W., Chen, P.J., and Chen, K.F. (2011). Signal transducer and activator of transcription 3 is a major kinase-independent target of sorafenib in hepatocellular carcinoma. J. Hepatol. *55*, 1041–1048.

24.. Su, J.C., Tseng, P.H., Wu, S.H., Hsu, C.Y., Tai, W.T., Li, Y.S., Chen, I.T., Liu, C.Y., Chen, K.F., and Shiau, C.W. (2014). SC-2001 overcomes STAT3-mediated sorafenib resistance through RFX-1/SHP-1 activation in hepatocellular carcinoma. Neoplasia *16*, 595–605.

25.. Zhao, W., Bai, B., Hong, Z., Zhang, X., and Zhou, B. (2020). Berbamine (BBM), a natural STAT3 inhibitor, synergistically enhances the antigrowth and proapoptotic effects of sorafenib on hepatocellular carcinoma cells. ACS Omega *5*, 24838–24847.

26.. Byeon, H.K., Ku, M., and Yang, J. (2019). Beyond EGFR inhibition: multilateral combat strategies to stop the progression of head and neck cancer. Exp. Mol. Med. *51*, 1–14.

27.. Kim, S., and Lee, J.W. (2014). Membrane proteins involved in epithelial-mesenchymal transition and tumor invasion: studies on TMPRSS4 and TM4SF5. Genomics Inform *12*, 12–20.

28.. Tsuchikama, K., and An, Z. (2018). Antibody-drug conjugates: recent advances in conjugation and linker chemistries. Protein Cell *9*, 33–46.

29.. June, C.H., O'Connor, R.S., Kawalekar, O.U., Ghassemi, S., and Milone, M.C. (2018). CAR T cell immunotherapy for human cancer. Science *359*, 1361–1365.

30.. Pettitt, D., Arshad, Z., Smith, J., Stanic, T., Hollander, G., and Brindley, D. (2018). CAR-T cells: a systematic review and mixed methods analysis of the clinical trial landscape. Mol. Ther. *26*, 342–353.

31.. Jung, C.R., Hwang, K.S., Yoo, J., Cho, W.K., Kim, J.M., Kim, W.H., and Im, D.S. (2006). E2-EPF UCP targets pVHL for degradation and associates with tumor growth and metastasis. Nat. Med. *12*, 809–816.

32.. Lee, Y., Yoon, J., Ko, D., Yu, M., Lee, S., and Kim, S. (2021). TMPRSS4 promotes cancer stem-like properties in prostate cancer cells through upregulation of SOX2 by SLUG and TWIST1. J. Exp. Clin. Cancer Res. *40*, 372.

33.. Lee, Y., Ko, D., Yoon, J., Lee, Y., and Kim, S. (2021). TMEM52B suppression promotes cancer cell survival and invasion through modulating E-cadherin stability and EGFR activity. J. Exp. Clin. Cancer Res. *40*, 58.

34.. Yang, H.Y., Kang, K.J., Chung, J.E., and Shim, H. (2009). Construction of a large synthetic human scFv library with six diversified CDRs and high functional diversity. Mol. Cells *27*, 225–235.

35.. Zhang, Y.F., and Ho, M. (2017). Humanization of rabbit monoclonal antibodies via grafting combined Kabat/IMGT/Paratome complementarity-determining regions: rationale and examples. MAbs *9*, 419–429.

36.. Gao, J., Aksoy, B.A., Dogrusoz, U., Dresdner, G., Gross, B., Sumer, S.O., Sun, Y., Jacobsen, A., Sinha, R., Larsson, E., et al. (2013). Integrative analysis of complex cancer genomics and clinical profiles using the cBioPortal. Sci. Signal. *6*, pl1.

37.. Cerami, E., Gao, J., Dogrusoz, U., Gross, B.E., Sumer, S.O., Aksoy, B.A., Jacobsen, A., Byrne, C.J., Heuer, M.L., Larsson, E., et al. (2012). The cBio cancer genomics portal: an open platform for exploring multidimensional cancer genomics data. Cancer Discov. *2*, 401–404.

OMTO, Volume *24*

Supplemental information

Therapeutic effects of TM4SF5-targeting chimeric and humanized monoclonal antibodies in hepatocellular and colon cancer models

Dongjoon Ko, Eunmi Kim, Eun-Ae Shin, Seo Hee Nam, Junghwa Yoon, Jin-Sook Lee, Yunhee Lee, Sora Park, Kyungsoo Ha, So-Young Choi, Jung Weon Lee, and Semi Kim

Supplementary Figure Legends

Supplementary Figure S1. (A) HT-29 cells were transfected with siRNA against TM4SF5 for 48 h and then immunostained with Ab27 (5 μg/ml) (green). Cell nuclei were counterstained with DAPI (blue). Scale bar, 50 μm. (B) Effect of STAT3 suppression on cell proliferation. Cells were transfected with siRNA against STAT3 for 48 or 72 h, lysed for immunoblot analysis to confirm suppression of STAT3 (upper), and subjected to cell proliferation assays (lower). Values represent means ± SD. $^{**}P < 0.01$; $^{***}P < 0.001$. (C) Scatter plots examining TM4SF5 mRNA expression (x-axis) and phosphorylated STAT3 at Tyr705 (y-axis) from liver hepatocellular carcinoma data (TCGA, PanCancer Atlas). Correlation was statistically analyzed using the Pearson test. The equation was automatically generated using the cBioPortal webpage tool.

Supplementary Figure S2. SNU449T$_7$-luc (stably overexpressing TM4SF5 and luciferase) cells (1×10^6) were injected subcutaneously into the backs of mice. On day 14, Ab27 (100 μg/mouse) was injected intraperitoneally at 2 or 3 day intervals for 2 weeks (total 7 times). (A) At 14 and 31 days after cell injection, bioluminescence images were saved. (B) Total bioluminescence flux over 2 week treatment was acquired using an IVIS Luminar imaging system. (C) Body weights of injected mice. Values represent means ± standard deviation (SD). $^{**}P < 0.01$.

Supplementary Figure S3. Ab27 inhibits colon cancer growth in a xenograft model. (A-C, E) HT-29 cells (2.5×10^6) were subcutaneously injected into the flanks of mice. Ab27 (142 μg/mouse) was i.p. injected into mouse (total of six injections). (A) Upper: Tumor volume

(length × width^2/2). Values of the maximum and minimum in each group were excluded from the mean calculation. Middle: Body weight of injected mice. Lower: Photos of tumor-bearing mice on day 33. (B) Ki67 staining of tumor sections was performed to measure the level of cell proliferation. Representative images are shown. Scale bar, 100 μm. (C) Cell death area was measured from (B) using ImageJ. Values represent means ± SD. *P < 0.05; **P < 0.01. P-value is shown on the graph (A). (D) HT-29 cells were incubated with Ab27 (25 μg/ml) for 48 h under suspension culture conditions, and then stained with annexin V and PI for flow cytometry. HA6 was used as a negative control antibody. (E) Immunoblot analysis of tumor extracts from (A). Densitometric quantification of bands on the immunoblot was performed using GAPDH as a loading control except that phosphorylated STAT3 and ERK1/2 were normalized against the corresponding total protein. Values of the maximum and minimum per group were excluded for the mean calculation. Values represent means ± SD. *P < 0.05; **P < 0.01.

Supplementary Figure S4. (A, B) Purified recombinant antigen proteins [human EC2-mouse Fc (hEC2-mFc) and mouse EC2-mouse Fc (mEC2-mFc)] (A) and purified Ab27 scFv-6×His-HA form (B) were analyzed by SDS-PAGE followed by Coomassie brilliant blue staining. R, reducing; NR, non-reducing condition. (C) Antigen-binding ELISA. Ninety-six-well immunoplates were coated with purified hEC2-mFc or mEC2-mFc (100 ng/well), and blocked with 2% BSA. Ab27 (scFv-6×His-HA form; serial diluted) was added, followed by HRP-conjugated anti-HA. Color was developed with TMB substrate solution and absorbance was measured at 450 nm.

Supplementary Figure S5. (A) Schematic representation of chimeric (Ab27) and humanized (Ab27-hz9) monoclonal antibodies of scFv-Fc format. C domain, constant domain; V domain, variable domain; VH, heavy chain variable domain; VL, light chain variable domain. (B) *In silico* immunogenicity analysis. Identification of immunogenic sequences was performed using the IEDB MHC II prediction server (Kim, Y., et al. (2012). Immune epitope database analysis resource. Nucleic Acids Res. 40, W525–W530), which employs a consensus approach combining NN-align, SMM-align, CombLib, and Sturniolo method. The results from IEDB prediction were visualized using the web-based analysis system in KBIOHealth (Osong, Korea). In the visualized results, colors indicate the degree of immunogenicity of an amino acid sequence, determined by the percentile rank. Immunogenic sequences on the right heat map are represented by 25%, 35%, and 50% cut-offs for low (P10, yellow), medium (P5, orange), and high (P1, red), respectively. VH, heavy chain variable domain; VL, light chain variable domain.

Supplementary Figure S6. Purified Ab27-hz9 was analyzed by SDS-PAGE, followed by Coomassie brilliant blue staining. Human IgG was loaded for comparison. R, reducing; NR, non-reducing condition.

Supplementary Figure S7. *In vivo* tumor targeting of Ab27 and Ab27-hz9 in an endogenous TM4SF5-expressing liver cancer xenograft. SNU-398 cells were subcutaneously injected into the flanks of nude mice to generate tumor-bearing mice. DyLight 755-labeled Ab27, Ab27-hz9, or normal human IgG was injected into the tail vein of tumor-bearing mice, and fluorescence was measured after 96 h. (A) Whole bodies of mice. (B)

Dissected mice at 96 h. Arrow indicates tumor mass. (C) Total fluorescence flux of tumor mass. Values represent means ± SD. $^{**}P < 0.01$.

Supplementary Figure S1. Ko et al.

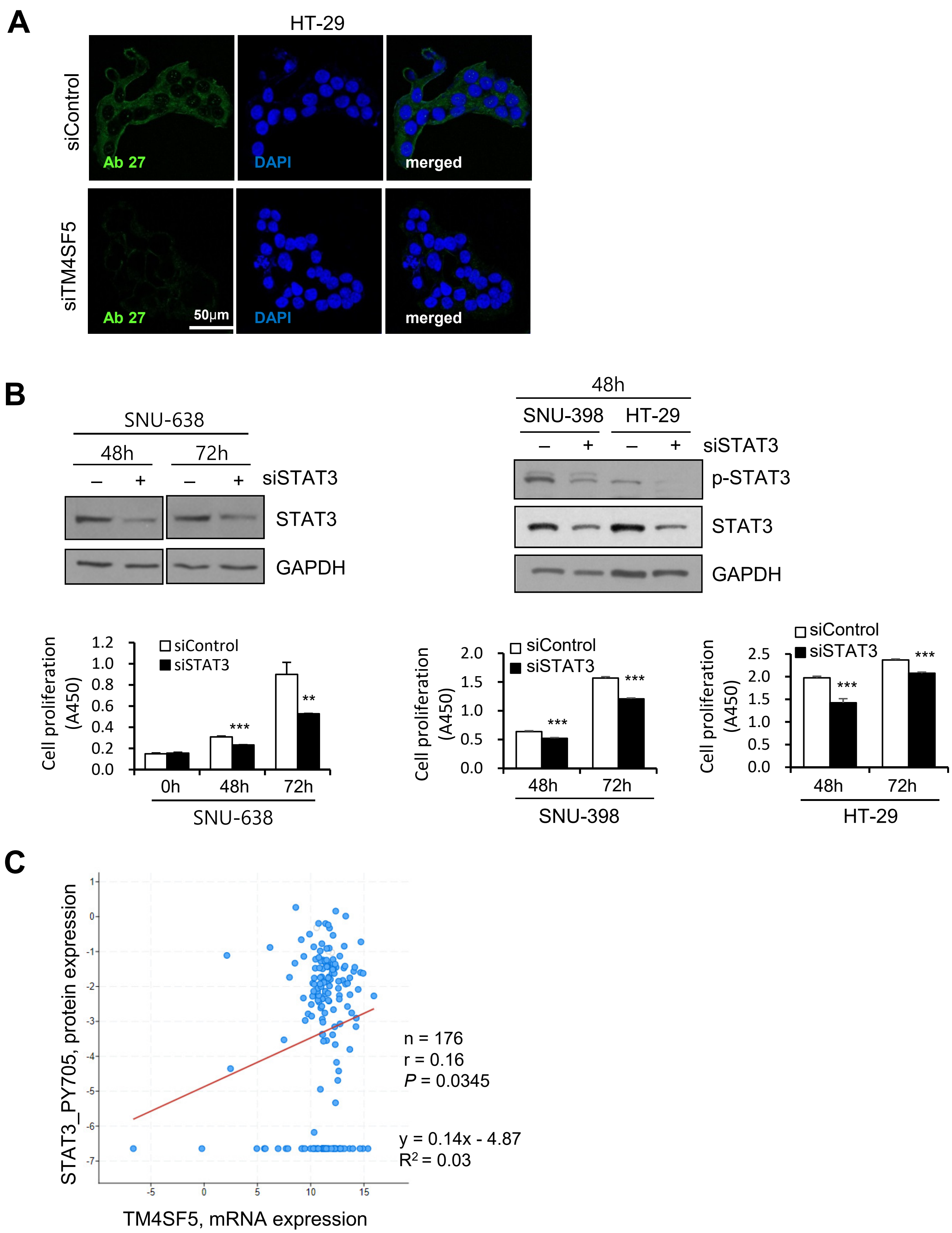

Supplementary Figure S2. Ko et al.

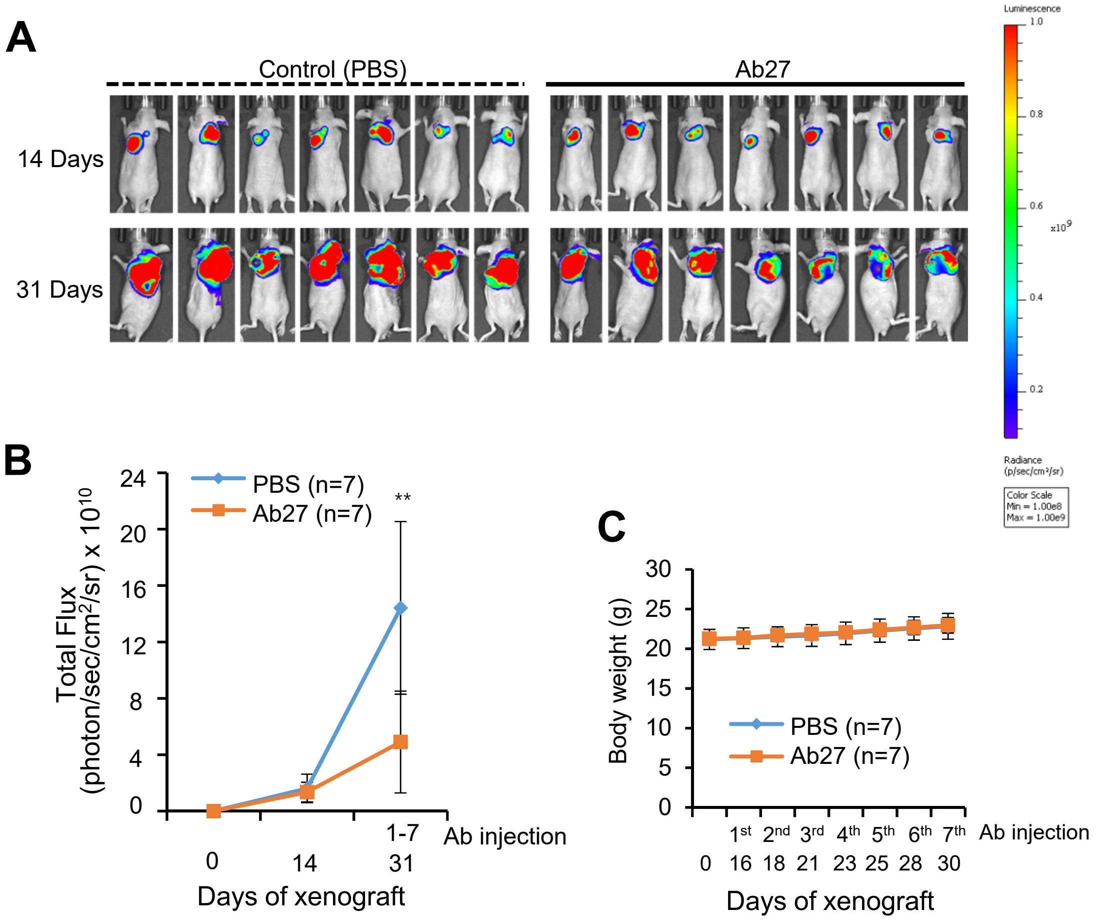

Supplementary Figure S3. Ko et al.

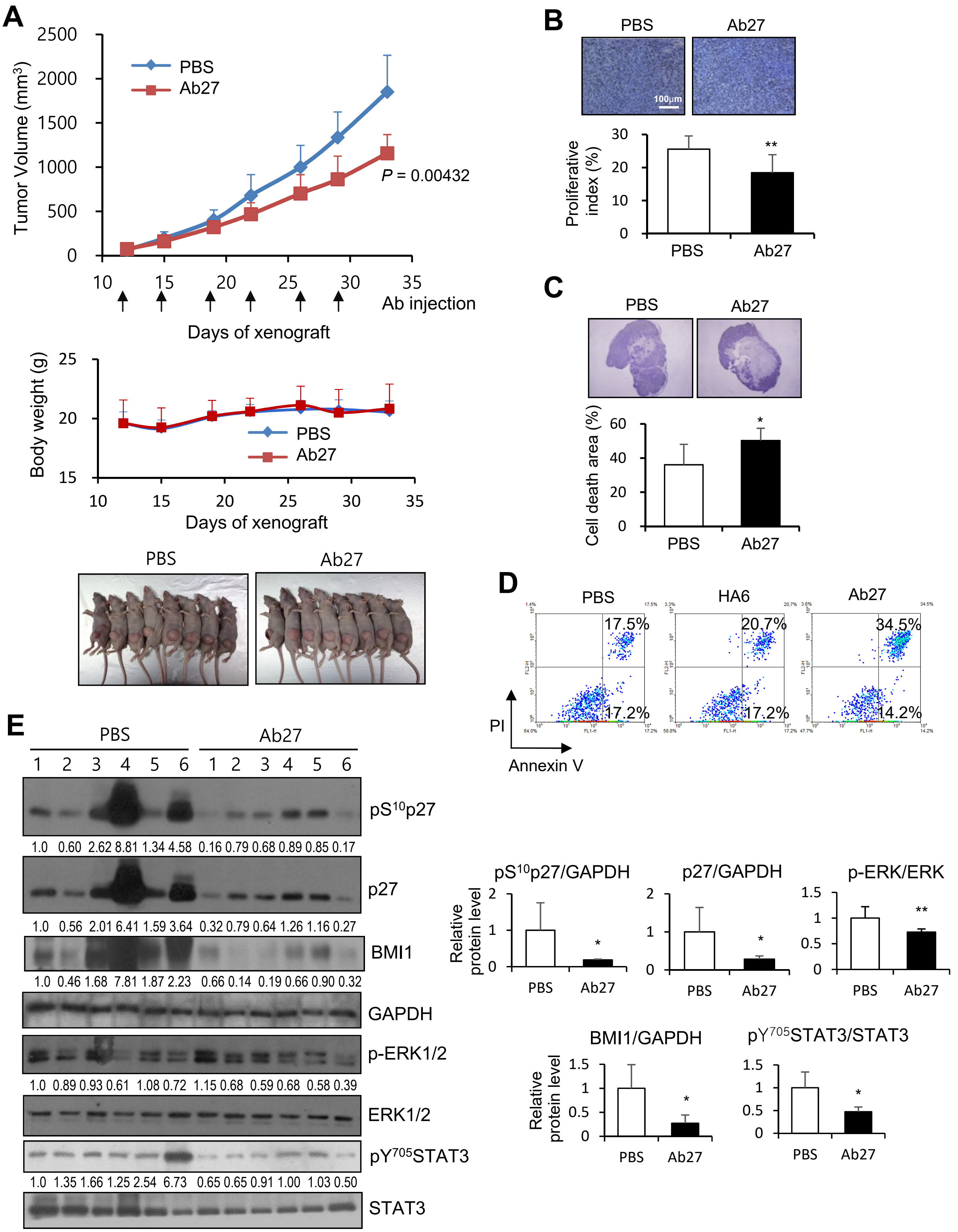

Supplementary Figure S4. Ko et al.

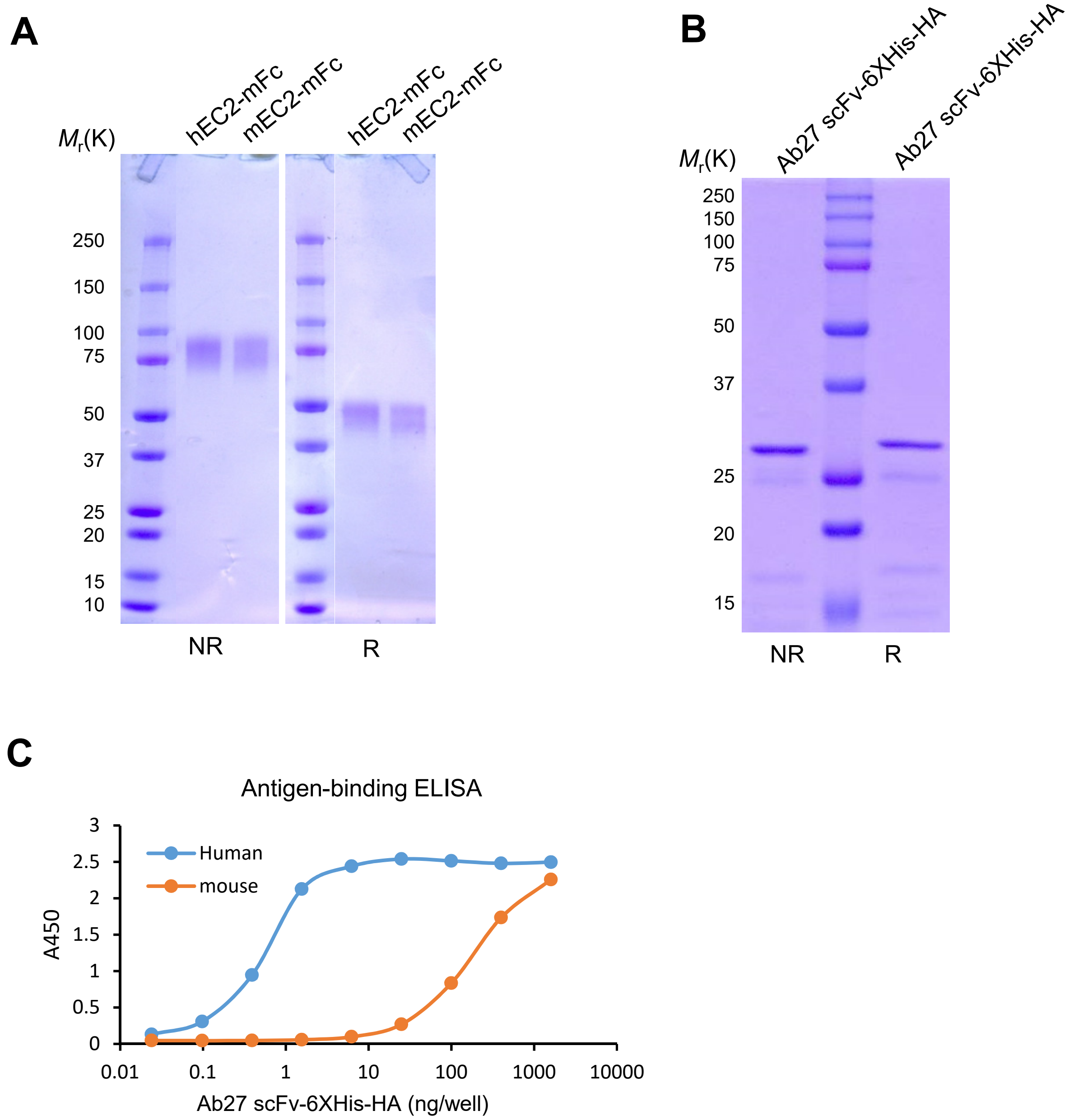

Supplementary Figure S5. Ko et al.

A

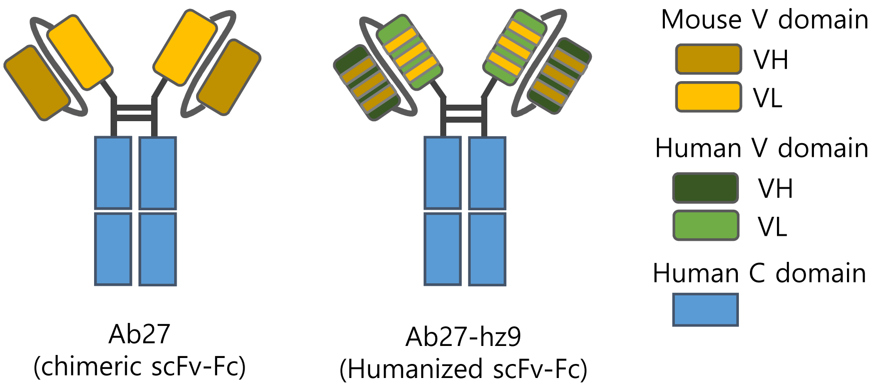

B

In silico immunogenicity analysis

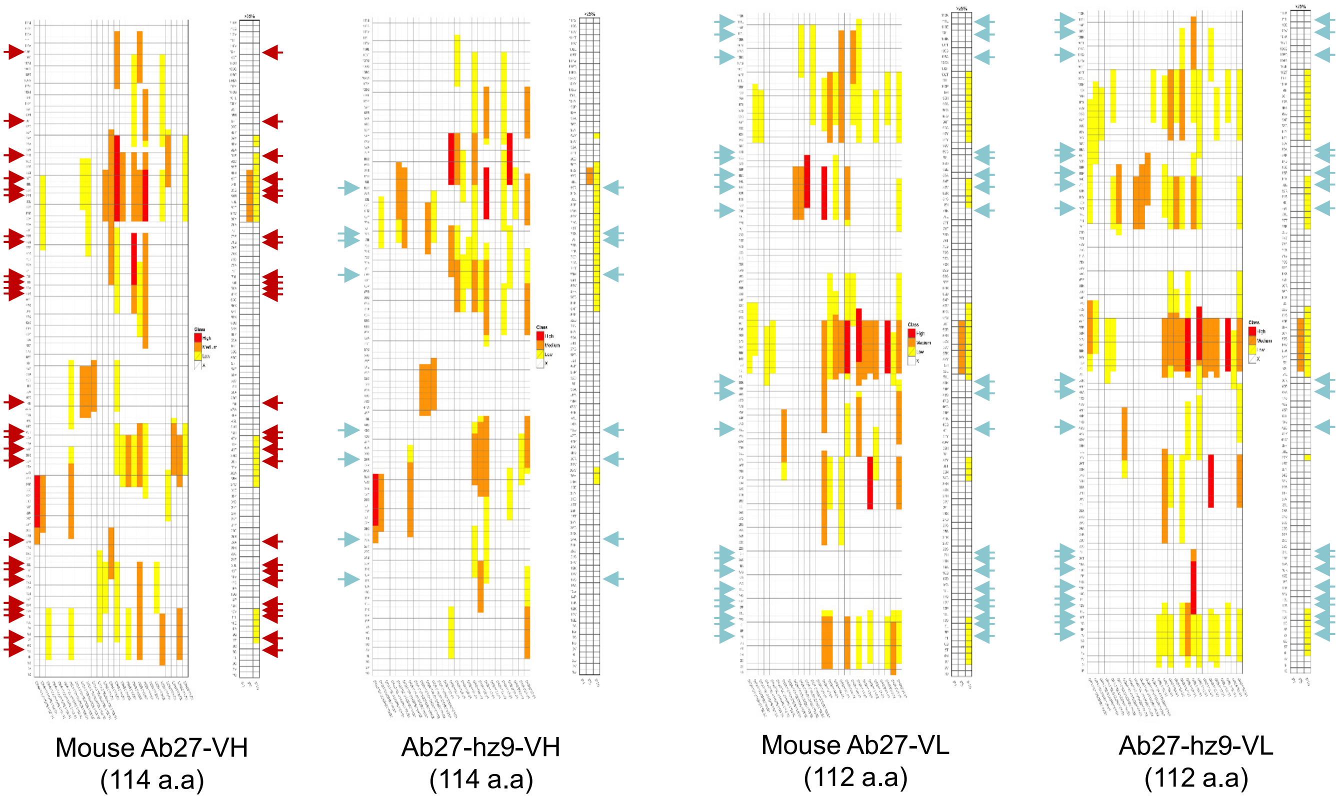

Supplementary Figure S6. Ko et al.

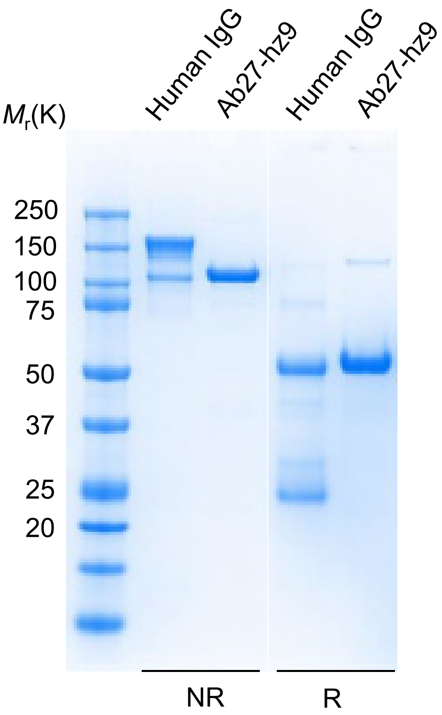

Supplementary Figure S7. Ko et al.

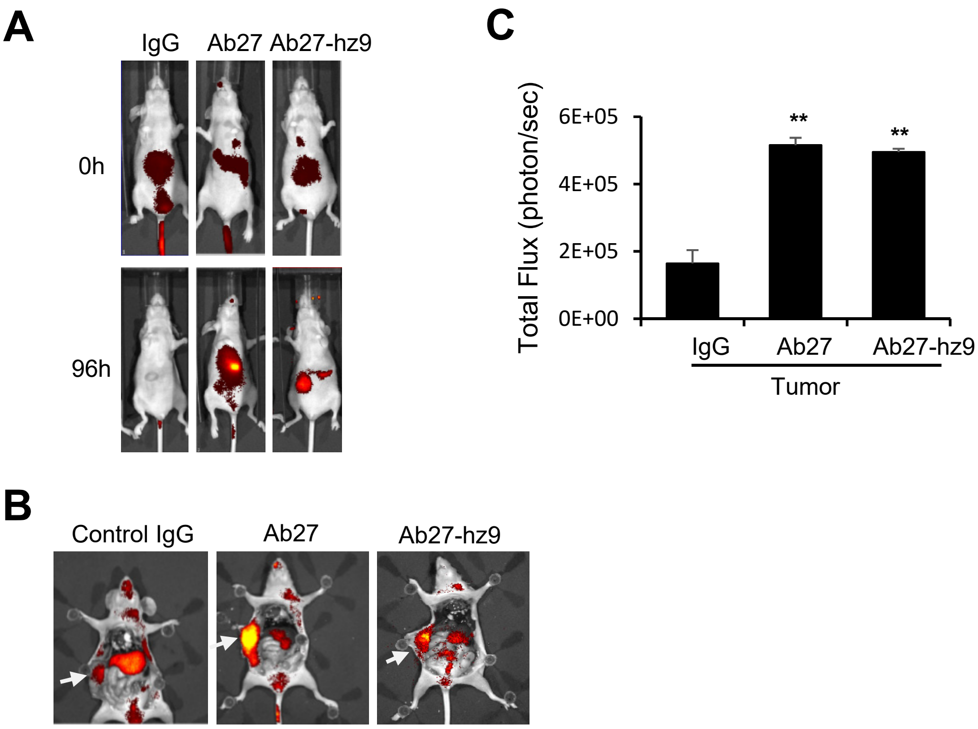

Supplement: Document S2. Article plus supplemental information [file mmc2.pdf]
